# Deciphering epigenomic code for cell differentiation using deep learning

**DOI:** 10.1186/s12864-019-6072-8

**Published:** 2019-09-12

**Authors:** Pengyu Ni, Zhengchang Su

**Affiliations:** Department of Bioinformatics and Genomics, The University of North Carolina at Charlotte, 9201 University City Boulevard, Charlotte, NC 28223 USA

**Keywords:** Deep learning, Histone modification, T cell differentiation

## Abstract

**Background:**

Although DNA sequence plays a crucial role in establishing the unique epigenome of a cell type, little is known about the sequence determinants that lead to the unique epigenomes of different cell types produced during cell differentiation. To fill this gap, we employed two types of deep convolutional neural networks (CNNs) constructed for each of differentially related cell types and for each of histone marks measured in the cells, to learn the sequence determinants of various histone modification patterns in each cell type.

**Results:**

We applied our models to four differentially related human CD_4_^+^ T cell types and six histone marks measured in each cell type. The cell models can accurately predict the histone marks in each cell type, while the mark models can also accurately predict the cell types based on a single mark. Sequence motifs learned by both the cell or mark models are highly similar to known binding motifs of transcription factors known to play important roles in CD_4_^+^ T cell differentiation. Both the unique histone mark patterns in each cell type and the different patterns of the same histone mark in different cell types are determined by a set of motifs with unique combinations. Interestingly, the level of sharing motifs learned in the different cell models reflects the lineage relationships of the cells, while the level of sharing motifs learned in the different histone mark models reflects their functional relationships. These models can also enable the prediction of the importance of learned motifs and their interactions in determining specific histone mark patterns in the cell types.

**Conclusion:**

Sequence determinants of various histone modification patterns in different cell types can be revealed by comparative analysis of motifs learned in the CNN models for multiple cell types and histone marks. The learned motifs are interpretable and may provide insights into the underlying molecular mechanisms of establishing the unique epigenomes in different cell types. Thus, our results support the hypothesis that DNA sequences ultimately determine the unique epigenomes of different cell types through their interactions with transcriptional factors, epigenome remodeling system and extracellular cues during cell differentiation.

**Electronic supplementary material:**

The online version of this article (10.1186/s12864-019-6072-8) contains supplementary material, which is available to authorized users.

## Background

Cell differentiation is achieved by the remodeling of the same genome that each cell inherits from the zygote. Genome remodeling involves alterations of methylation of certain cytosine residues in the genomic DNA and changes in various covalent modifications of histones in the nucleosomes, conferring a unique epigenome to each resulting cell type that expresses a unique set of gene products [1]. Increasing lines of evidence have suggested that the epigenome in a cell type is established step-wisely along the developmental lineage through the interplay of genomic sequence, chromatin remodeling systems and extracellular environmental cues [2–5]. As the latter two factors are the results of interactions of the products of genomic sequences, the epigenome of a cell type is ultimately determined by the genomic sequence that recruits the chromatin remodeling systems [6–9]. For example, in a recent study, Whitaker and colleagues [8] have shown that short DNA motifs enriched in the epigenetically modified genomic regions could predict the specific histone modifications in specific cell types using a random forest-based method. However, this method could not discover sequence determinants ab initio because pre-selected motifs were needed to train the models. Therefore, new methods are needed to gain a better understanding of the sequence determinants that specify the unique epigenome of each cell type produced during cell differentiation.

Recent progress in machine-learning has demonstrated that deep convolutional neural networks (CNNs) can achieve very high accuracy in predicting transcription factor (TF) binding affinity [10] and epigenetic marks in various cell types [11–13]. Unlike traditional neural networks, the kernels in the convolutional layers in a CNN can automatically learn the features of the objects (i.e., the sequence motifs in epigenetically modified regions), and thus the learned features can provide insights into the underlying mechanisms of the modeling systems. Although efforts have been made to explain the learned motifs in epigenetically modified regions in biological contexts types [11–13], the mixed CNN models employed in these earlier studies lack the power of comparison, limiting their ability to explain the learned motifs for their roles in determining the unique epigenetic modification patterns in different cell types. To overcome these shortcomings, we developed two types highly accurate CNN models to facilitate the explanation of the learned motifs: the cell type model to predict different histone marks in a given cell by learning motifs that specify the histone marks in the cell type, and the histone mark model to predict different cell types by learning motifs that determine different patterns of a given histone mark in different cell types. To evaluate the capability of the models to learn the histone mark-determining motifs, we applied them to a dataset of six histone marks obtained in four human CD_4_^+^ T cell types produced at different stages of cell differentiation [14], i.e., the native T (Tn) cells, central memory T (Tcm) cells, T effector memory (Tem) cells and CD_4_^+^ terminally differentiated CD_45_RA^+^ memory (Temra). The relatively rich knowledge about the regulators and the differentiation process of these T cell subpopulations could facilitate the validation of predictions. Indeed, we found that many sequence motifs learned in the CNN models of both the cell types and histone modifications are highly similar to known binding motifs of TFs known to play important roles in CD_4_^+^ T cell differentiation. Intriguingly, the shared motifs learned in different cell models support the linear model of CD_4_^+^ T cell development, consistent with the earlier results based on the patterns of changes in DNA methylation and DNase accessibility of the genome as well as transcriptomes in the cells [14], while the shared motifs learned in different histone mark models reflect the functional relationships of the marks. Furthermore, by computing the s of the learned motifs on the prediction of the CNNs, we were able to pinpoint specific roles and interactions of their cognate TFs in determining unique histone modification patterns in different cell types, thereby providing new insights into the underlying mechanisms of histone modifications during cell differentiation.

## Results

### The cell type CNN models are highly accurate and robust for predicting various histone modifications in different cell types

**Fig. 1:**
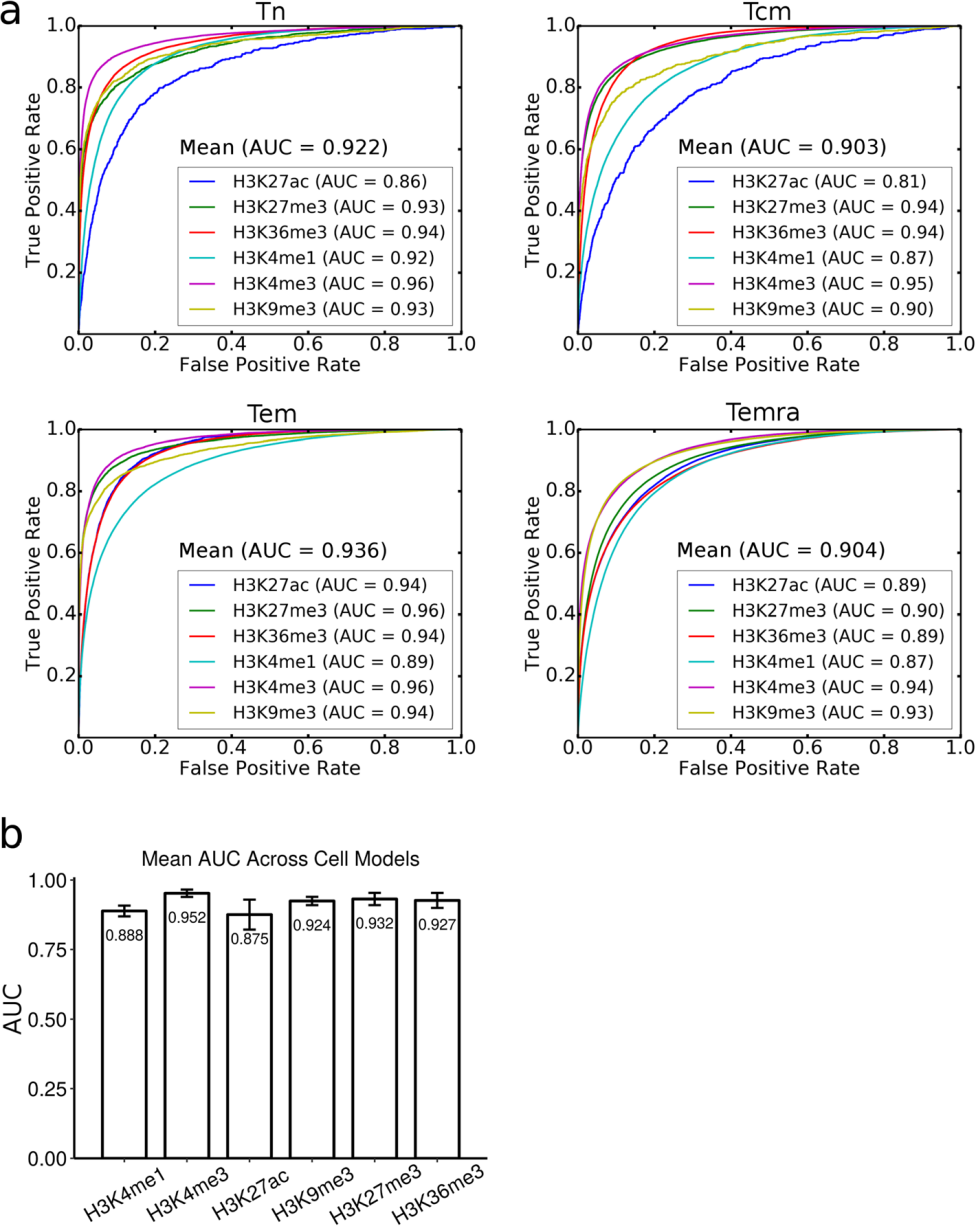
Performance of the CNN models of the four cell types for predicting the six histone marks. **a**. The ROCs of the Tn, Tcm, Tem and Temra cell models for predicting the six histone marks. **b**. Mean AUC for each histone mark across the four cell type models

In the genome of a cell type, different loci are modified by the same and/or different chromatin marks in unique ways. It is the different combinations of these chromatin marks that determine the distinct chromatin states of the genomes in different cell types [15]. To learn the sequence determinants that govern the unique combinations of histone modifications in a cell type, we constructed a CNN model for the cell type for predicting the histone marks in its genome. We first evaluated the model using the data set of six histone marks collected from the four human CD_4_^+^ T cell types derived during T-cell differentiation [14]. Specifically, we used 459,814, 653,272, 978,543 and 2,131,540 histone modification peaks in building the models for the Tn, Tcm, Tem and Temra cells, respectively (Methods). As shown in Fig. 1a, all the models perform very well for predicting the patterns of the six histone marks in each of the four cell types, with an average accuracy and AUC (area under the receiver operating characteristic (ROC) curve) of 91.53% and 0.916, respectively, which are better than the results achieved by the earlier state-of-the-art CNN models for the same marks although their results were based on a different dataset [11].

To evaluate the generality and robustness of our model, we applied it to a dataset for the six histone marks collected from the H1 human embryonic stem (H1) cells, trophoblast-like (TBL) cells, mesendoderm (ME) cells, mesenchymal (MSC) cells and neural progenitor (NPC) cells [16], which has been studied intensively [8]. In this case, we used 1,038,201, 363,349, 880,462, 1,011,252, and 315,266 histone modification peaks in building the models for the H1, TBL, ME, MSC and NPC cells. As show in Additional file 1: Figure S1A, the models also perform very well for predicting the patterns of the six histone marks in the five cell types, with an average accuracy 90.6% and AUC 0.917, which are comparable to those obtained in the CD_4_^+^ cell dataset (91.53% and 0.916), but also are better than the results achieved by the earlier state-of-the-art CNN models for the same markers albeit on a different dataset (AUC 0.856) [11]. Our models (average accuracy 90.6% and AUC 0.917) also outperform the earlier random forest-based algorithm on the same dataset (average accuracy 79.0%, average AUC 0.837, Additional file 1: Figure S1B). The relative performance of our models on predicting the six marks also is consistent with the random forest-based method (Additional file 1: Figure S1B) except for H3K9me3, which holds the second place in our model while it was ranked fifth in the earlier study. Such consistent performance of the different methods in different datasets strongly suggests that the active enhancer marks H3K27ac (AUC 0.880) and H3K4me1 are more complicatedly used in the cell types than the other marks. Therefore, our cell type CNN models are very robust and highly accurate for predicting unique patterns of various histone marks in given different cell types.

### The histone mark CNN models are highly accurate and robust for predicting different cell types based on a histone mark

To reveal the determinants that specify different patterns of the same histone mark in different cell types, we constructed a CNN model for each histone mark for predicting different cell types based on the different patterns of the same mark. We also first evaluated the accuracy of the models using the dataset collected from the four CD_4_^+^ T cell types [14], and employed 227,420, 691,032, 839,057, 867,398, 296,079 and 435,351 histone peaks in building the models for H3K27ac, H3K27me3, H3K36me3, H3K4me1, H3K4me3 and H3K9me3, respectively (Methods). As shown in Additional file 1: Figure S2A and B, the models generally perform very well for predicting each cell type, although the models for the gene repression-related mark H3K27me3 (AUC 0.95) and the heterochromatin-related mark H3K9me3 (AUC 0.93) perform better than the models for the activation-related marks H3K36me3 (AUC 0.87), H3K27ac (AUC 0.85), H3K4me3 (AUC 0.83) and H3K4me1 (AUC 0.71).

To evaluate the generality and robustness of the mark model, we also applied it to the dataset collected from the human embryonic cells H1 and four of its derived types [16], and used 332,704, 458,844, 952,615, 185,182, 253,289, 360,040 histone modification peaks in building the models for H3K4me1, H3K9me3, H3K36me3, H3K4me3, H3K27me3 and H3K27ac, respectively (Methods). As shown in Additional file 1: Figures S3A and B, similar to the results from the CD_4_^+^ T cell dataset (Additional file 1: Figure S2A and B), the models also generally perform very well, although the models for the gene repression-related mark H3K27me3(AUC 0.909) and the heterochromatin-related mark H3K9me3(AUC 0.862) perform better than the models for the activation-related H3K4me3 (AUC 0.815), H3K4me1 (AUC 0.720), H3K27ac (AUC 0.770) and H3K36me3 (AUC 0.679). These consistent results from different datasets from different sources strongly suggest that the two repressive histone marks are more distinctly used in different cell types than the four activation-related marks. Therefore, our histone mark CNN models are highly accurate and very robust for predicting different cell types based on the pattern of single histone marks.

### Patterns of different histone marks in a cell type as well as different patterns of the same histone mark in different cell types are largely determined by a unique set of motifs

**Fig. 2:**
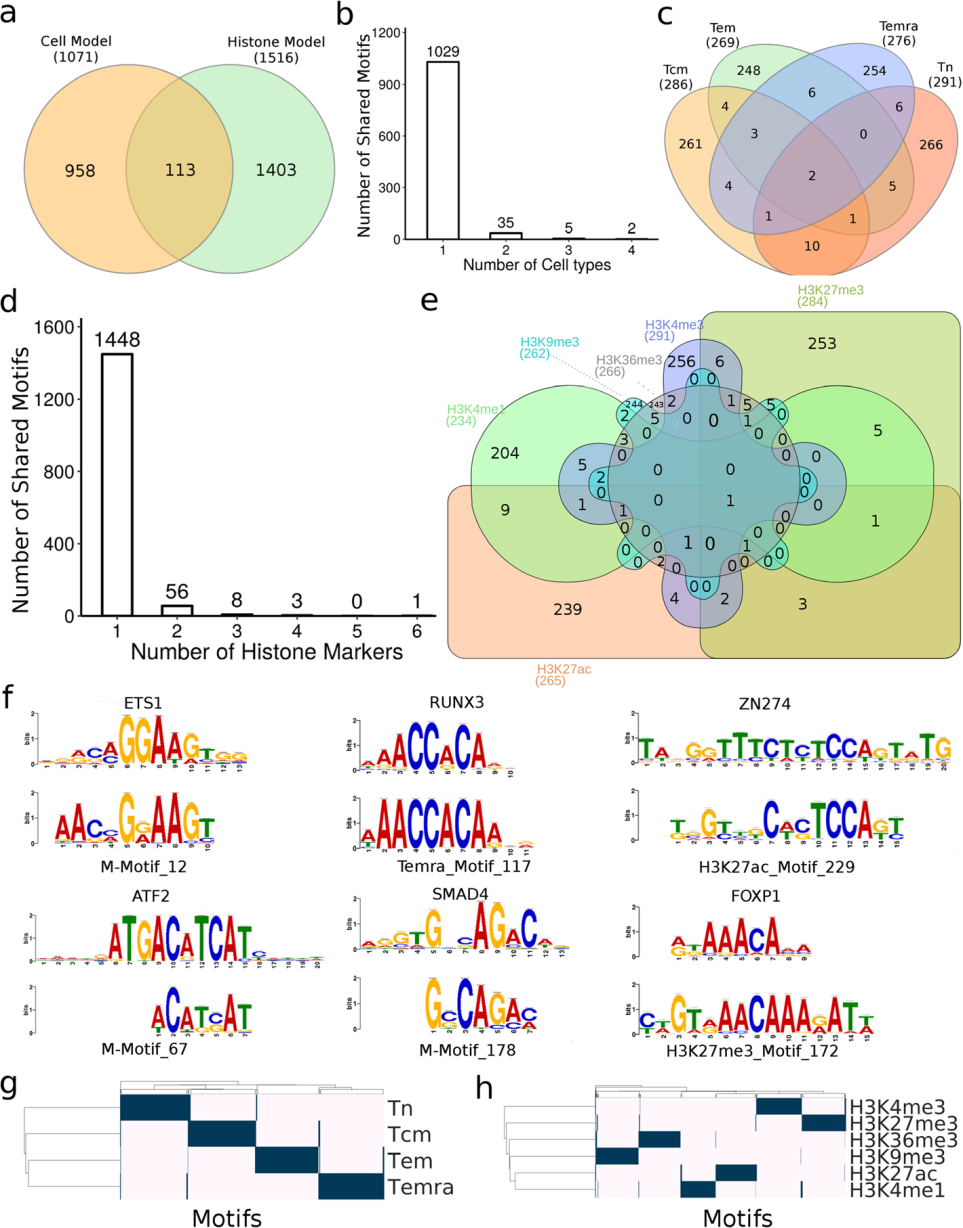
Known and novel TF binding motifs learned in the first convolution layers of the CNN models. **a**. Overlap of motifs learned in the cell models and histone mark models. **b**. Number of learned motifs shared by different number of cell models. **c**. Venn diagram showing the number of learned motifs shared by the cell models. **d**. Number of learned motifs shared by different number of histone mark models. **e**. Venn diagram showing the number of learned motifs shared by the histone mark models. **f**. Examples of learned motifs matching known motifs involved in T cell functions. **g**, **h**. Hierarchical two-way clustering of the cells and histone marks, respectively, based on the similarity of the learned motifs profiles in the models using hamming distance and average linkage. The Venn diagrams were drew using INteractiVenn [17]

The superior performance of our cell models indicates that the filters in the convolutional layers have largely learned the sequence determinants for specifying the patterns of various histone marks in the cell type; while the superior performance of our histone mark models suggest that the filters in the convolutional layers have largely learned the sequence determinants for governing different patterns of the same histone mark in different cell types. These results promoted us to reveal these sequence determinants by looking into the filters in the convolutional layers of the models. In particular, we expect that the filters in the first convolutional layer may have learned the binding motifs of TFs involved in the specification of different histone modification patterns in different cell types. In other words, these filters may correspond to position weight matrices (PWMs) of the TF binding motifs. To this end, we constructed motif models for all the filters learned in the first constitutional layers, resulting in 295, 295, 278 and 285 motifs in the Tn, Tcm, Tem and Temra cell models, respectively; and 280, 291, 271, 270, 293, 267 motifs for the H3K27ac, H3K27me3, H3K36me3, H3K4me1, H3K4me3, H3K9me3 mark models, respectively. Some of the motifs learned in different models are highly similar to each other, thus we clustered them according to their similarity (Methods), resulting in 2474 clusters. Of these clusters, 203 are formed by more than two learned motifs, and we call each of them a Merged Motif (M-Motif), while the remaining 2271 are singleton motifs, and we consider each of them as a cell- or mark-specific motif dependent on the type of the model by which it is learned. Interestingly, 113 (4.57%) of these 2474 unique motifs are shared by at least a cell type model and a histone mark model, indicating that common sequence determinants were captured by the two types of models. On the other hand, the remaining 958 and 1403 motifs are unique to the cell type models and histone mark models, respectively (Fig. 2a). Thus, besides the common motifs, both the cell type models and mark models captured quite different sets of motifs for predicting the patterns of different histone modifications in the cells and the cell types based on single histone marks, respectively. Furthermore, 42 (3.92%) of the 1071 motifs learned in the cell type models and 68 (4.49%) of the 1516 motifs learned in the histone mark models are shared by more than two cell models (42/1071 = 3.92%) and two mark models (68/1516 = 4.49%) (Fig. 2b~e), respectively. However, only two (0.21%) and one (0.10%) motifs are shared by all the four cell type models and all the six histone mark models, respectively. The remaining 1029 (96.08%) and 1448 (95.51%) motifs are unique to a single cell type model and a single mark model, respectively. These results suggest that the unique patterns of various histone marks in each cell type as well as the different patterns of the same histone mark in different cell types are largely determined by a unique set of motifs, although they may share some common ones. This conclusion agrees with the general understanding about how the unique epigenomes are established in different cells type by the interplay of TF, chromatin remodeling systems and environment cues [2–5].

At an E-value threshold of 0.5, 974 (39.37%) of the 2474 motifs match known human TF binding motifs in the HOCOMOCO database [18], and many of them are known to be involved in T cell differentiation (Fig. 2f). We described a few examples of them. M-Motif 12 shared by all the cell type models matches that of ETS1 that controls T cell differentiation by regulating the expression of signaling molecules [19, 20] in response to external environment stimuli. M-Motif 67 shared by the H3K9me3 and Tem models matches that of ATF2 that is an histone acetyltransferase for histones H2B and H4, playing an essential role in the T cells activation in late-stage [21, 22]. Temra-Motif 117 learned in the Temra model matches that of RUNX3, which plays a crucial role in T cell’s differentiation by interacting with master regulators cooperatively [23]. M-Motif 178 shared by the Tn and H3K4me1 models resembles that of SMAD4 that cooperatively regulates interleukin 2 receptor in T cells and balances the differentiation of CD_4_^+^ T cells [24, 25]. H3K27ac-Motif 229 learned in the H3K27ac model matches that of ZN274 that is involved in transcription repression [26]. H3K27me3-Motif 127 learned in the H3K27me3 model resembles that of FOXP1, which is the “naive keeper” for T memory cell differentiation [14, 27]. These results suggest that at least 39.37% of the learned motifs that match known ones are likely to be authentic motifs of the cognate TFs.

### Motifs learned in the cell type models reflect the lineage of the cells

It is now well established that along the lineage of cell differentiation, the epigenomes of cells undergo step-wise changes with each cell division through the regulation of a specific set of both common and unique TFs in the derived intermediate and terminal cell types [2–5, 28, 29]. Cells in adjacent differentiation stages possess more similar epigenomes [10], presumably because they share more TFs than those that are distal from each other along the lineage of differentiation. To see whether this is reflected in the motifs learned by the cell type models, we hierarchically clustered the cell types based on the similarity of the learned motif profiles in the cell type models. As shown in Fig. 2g, Tn branches earliest in the tree while the three memory/effector T cell types form a clade, indicating that Tn is most distinct from the more developed cell types as generally believed. Tem and Temra form a clade, indicating that they are more similar to each other than to Tcm, which is in agreement with early observations [30]. These results suggest a linear lineage model of the development of these cells: Tn → Tcm → Tem → Temra, which is in line with the results derived based on changes in the DNA methylation, gene expression and DNAase accessibility in these cells [14]. Therefore, the sequence motifs learned in the cell type models indeed reflect the lineage relationships of the cells. It is highly likely that the unique motifs to a cell model account for the distinction of the cell type from the other cell types, while the shared motifs are responsible for the shared features of linearly closely-related cell types.

### Motifs learned in histone mark models reflect functional relationships of the marks

It is well-known that certain types of sequences can be co-modified by different histone marks, while other types of sequences tend to be exclusively modified by a specific mark [31]. To see whether such co-modifications and exclusiveness of the marks are reflected by the learned motifs in the histone mark models, we hierarchically clustered the histone marks based on the similarity of the learned motif profiles. As shown in Fig. 2h, H3K4me1 and H3K27ac form a group, which is consistent with the fact that they co-mark active enhancers, thus the respective modification systems might be recruited by some common motifs or similar mechanisms. On the other hand, H3K9me3, H3K27me3, K3K36m3 and H3K4me3 form a singleton group by themselves, which is consistent with the facts that they exclusively mark DNA domains with different epigenomic states [32]. For instance, H3K9me3 marks heterochromatins, H3K27me3 labels polycomb-associated domains, K3K36m3 marks transcribed gene body and H3K4me3 labels active promoters. Therefore, the learned motifs in the histone mark models indeed reflect the known functional relationships of the marks.

### The learned motifs have varying inferences on the prediction accuracy of the models

**Fig. 3:**
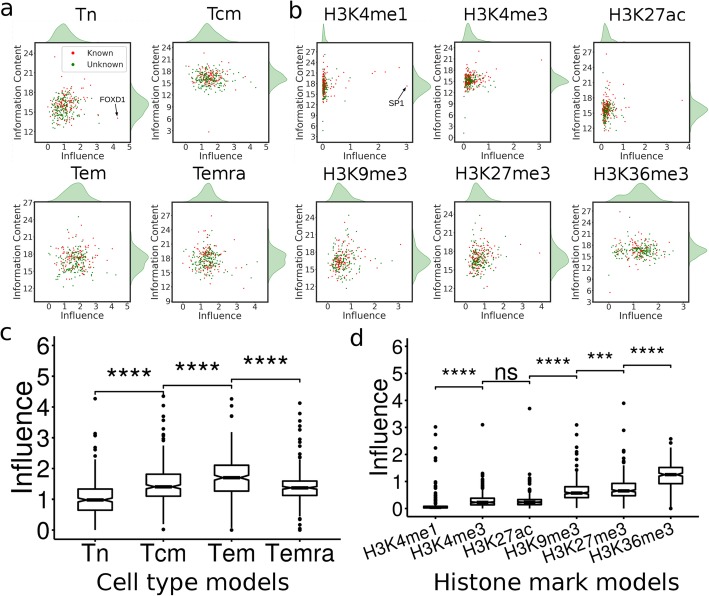
Distributions of the inference scores of learned motifs in cell type and histone mark models. **a**, **b.** Relationship between the inference scores and information contents of motifs learned in the four cell models and six histone mark models, respectively. **c**, **d**. Boxplots of the inference scores of the motifs learned in the cell models and histone mark models, respectively (***, *p* < 0.001; ****p < 0.0001; Wilcoxon test)

To evaluate the contribution and importance of a learned motif to the prediction of a model, we nullified the motif and then calculated its inference score on the predictions (Methods). The inference scores of the motifs learned in both the cell type models (Fig. 3a) and the histone mark models (Fig. 3b) have bell-shape distributions with different extent of right skewness. These results suggest that most learned motifs have intermediate inferences, while a small portion have large inferences on predicting the patterns of different histone marks in a cell type or different cell types based on single histone marks. The motifs with high influences might play crucial roles in the cell differentiation process. For example, in the Tn model, the motif with the highest influence score 4.26 (Fig. 3a) resembles that of FOXD1 that is involved in T cell proliferation [33]; in the H3K4me1 model, the motif with the highest inference score 2.74(Fig. 3b) resembles that of SP1 that plays a role in T cell differentiation [34]. The inferences of the motifs learned in either the cell type models (Fig. 3a) or the histone mark models (Fig. 3b) do not significantly correlate with their information contents, suggesting that only few positions of the motifs have a strong predictive power, which is consistent with the general understanding about the mechanisms of TF-DNA interactions. The learned motifs that do not match known motifs have similar inference scores to those matching known motifs (Fig. 3a and b), indicating that they are equally likely to be true motifs, and the unmatched ones are likely to be novel motifs of unknown TFs.

Interestingly, the inferences of motifs learned in Tn, Tcm, Tem cell models increased along the proposed linear cell lineage, and then decreased in the Temra cell model (Fig. 3c). These results suggest that the functions of learned motifs become more and more specific in determining the patterns of various histone modifications in the cells along the differentiation lineage Tn → Tcm → Tem, and then somehow become less specific in Temra. Furthermore, the inference scores of motifs learned in the six histone mark models are also significantly different from one another (Fig. 3d). Specifically, motifs learned in the models of H3K4me1, H3K27ac and H3K4me3 that mark active enhancers and promoters have the lowest inference scores, while those learned in the models of H3K9me3 and H3K27me3 that are associated with repression regions have the moderate inference scores, and those learned in the model of H3K36me3 that marks actively transcribed regions have the highest inference scores (Fig. 3b). These results suggest that the motifs specifying histone modifications in actively transcribed regions have the highest specificity, followed by those for determining histone modifications in repression regions, active promoters and enhancers regions.

### The motifs learned in a cell type model have highly variable inferences on different histone marks

**Fig. 4:**
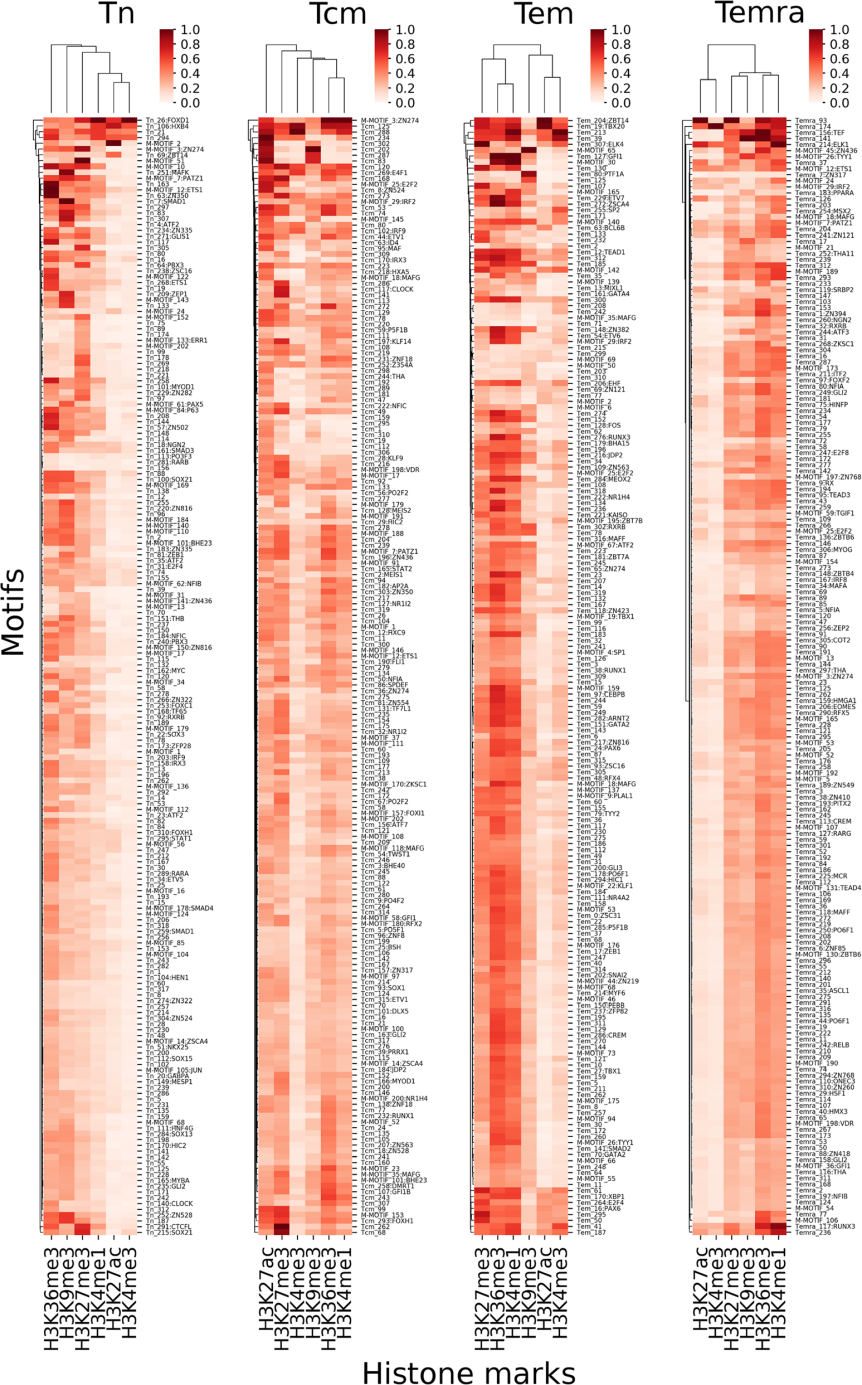
Inferences of the learned motifs on the prediction of each histone mark by the cell type models. The heatmaps show the influence scores of the top 100 learned motifs on predicting the six histone marks in the indicated cell type models. The scale bar shows range of the inference score of a motif on a histone mark

An important question in epigenomics study is to understand how different histone marks are placed at specific domains of the genome in a cell type. Our cell models might provide an easy way to address this question by simply finding out the learned motifs that impose a high inference on the prediction of each histone mark in the models. More specifically, we computed an inference score of each learned motif on each histone mark in a cell type model. Shown in Fig. 4 are the results for the learned motifs that are ranked top 100 for their inferences on predicting at least one histone mark in the cell type models. Clearly, the motifs learned in each cell type model have highly variable inferences on different histone marks. For example, in all the four cell type models, H3K36me3 and H3K27me3 are highly impacted by a large number of the learned motifs, while H3K4me3 is only highly impacted by a few learned motifs, such as Tn-26:FOXD1, Tn-106:HXB4, TN-21 and Tn- 294 in Tn (Fig. 4). H3K27ac is highly impacted by a large number of learned motifs in Tcm, but is highly impacted by only a few learned motifs in Tn, Tem and Temra. H3K4me1 is highly impacted by a larger number of learned motifs in Tcm, Tem and Temra, but is highly impacted by a few learned motifs in Tn. H3K9me3 is highly impacted by an intermediate number of learned motifs in all the four cell types. Moreover, in all the four cell models, only a few learned motifs have high inferences on all the histone marks, while most motifs have a high inference only on 1–3 histone marks (Fig. 4). For instance, in Tn model, only motifs Tn-26:FOXD1, Tn-106:HXB4, Tn-21 and Tn-294 have high inferences on all the six histone marks, while most of other motifs have high inferences only on one or two histone marks. Thus, each histone mark is impacted by a unique combination of motifs that may have inferences on more than two histone marks. These results suggest that the cognate TFs of most learned motifs exerting more specific inferences on one or two histone marks might play crucial roles in specifying the unique patterns of different histone marks in the cell type, while the cognate TFs of a few learned motifs having high inferences on multiple histone marks might be involved in the establishment of multiple histone marks, probably by playing roles in the common mechanisms of different histone modifications such as opening up of DNA domains.

### The motifs learned in a histone mark model have highly variable inferences on different cell types

Another important question in epigenomics study is to understand how the same histone mark is differentially placed in the genomes of different cell types. Our histone mark models might provide a convenient way to tackle this question by simply identifying the learned motifs that impose a high inference on the prediction of each cell type by the models. More specifically, we calculated an inference score of each learned motif on the prediction of each cell type by a histone mark model. Shown in Additional file 1: Figure S4 is the result of the motifs that are ranked top 100 for their inferences on predicting at least one cell type by the six histone mark models. Interestingly, motifs learned in each histone mark model have highly variable inferences on different cell types. For instance, in the H3K4me1 model, most of the learned motifs have similarly small inferences on all the four cell types, only few have high inferences on at least one cell type. However, the latter set of motifs exert high inferences only on one or two cell types with the exception that motif H3K4me1–236:HXC10 has high inferences on all the four cell types. Thus, it seems that H3K4me1 in each cell type is specified by a small set of motifs with unique combinations. In both the H3K4me3 and H3K27ac models, most of the learned motif have similarly small inferences on the Tem, Tcm and Tn cell types, only few have high inferences on at least one of these three cells types, suggesting that these two histone marks are specified by a small set of motifs with unique combination in these three cell types. However, most of the motifs learned in the H3K4me1 and H3K27ac models impose high inferences on the Temra cells, suggesting that these cells might have more complex H3K4me3 and H3K27ac modifications than the other three cell types, which is in line with the fact that Temra is the terminally differentiated cells with more activated enhancers and promoters. In the H3K9me3 and H3K27me3 models, each cell type is impacted by a large number of learned motifs with few having high inferences on more than three cell types, suggesting these two histone modifications in each cell type are specified by a large set of motifs with unique combinations. This result might be related to the functions of H3K9me3 that marks heterochromatins and of H3K27me3 that labels polycomb-associated domains. In the H3K36me3 model, the numbers of learned motifs having high inferences in the cells increase along their linear lineage: Tn → Tcm → Tem → Temra. Each cell type is highly impacted by a large number of the learned motifs that impact adjacent cells along the lineage. These results reflect the similarity of the transcriptomes of these adjacent cell types [8], and thus are in excellent agreement with the functions of H3K36me3 that marks actively transcribed genes. Taken together, the cognate TFs of few learned motifs that exert high inferences on multiple cell types might account for the similar patterns of a histone mark and the common mechanisms of the histone modification in different cell types, while the cognate TFs of the motifs that have more specific inferences might play crucial roles in specifying the different patterns of the histone modification in different cell types.

### Conserved learned motifs tend to have higher inferences on the predictions

**Fig. 5:**
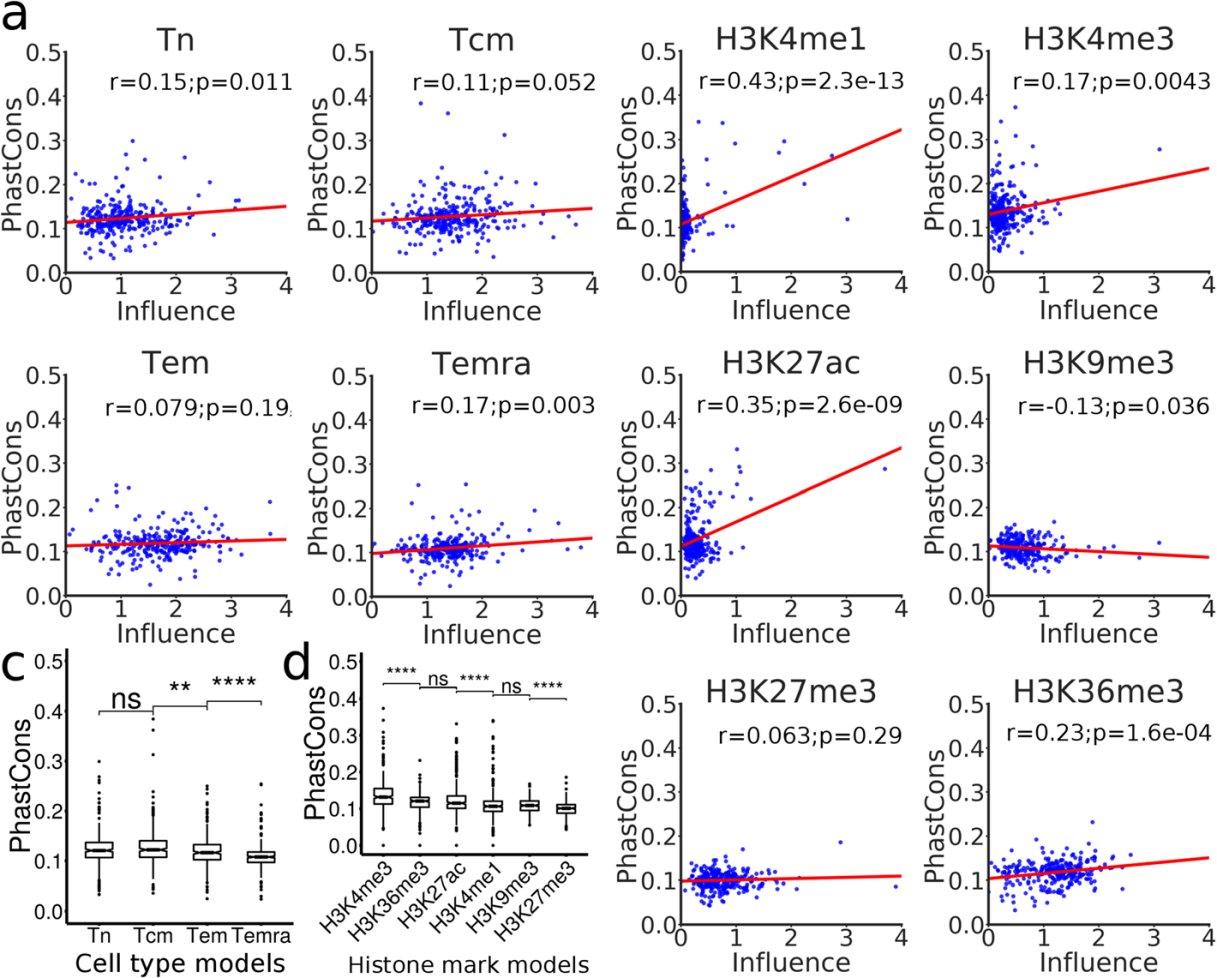
Relationship between the inference scores and PhastCons scores of the learned motifs. **a**, **b**. Relationship between the inference scores and PhastCons scores of the learned motifs in the cell models and the histone mark models, respectively. The red line is the linear regression between the inference scores and PhastCons scores. **c**, **d.** Boxplots of the PhastCons scores of the motifs learned in cells models and histone models, respectively (**, *p* < 0.01; ****, p < 0.0001; Wilcoxon test)

We also examined the relationships between the inference scores and the conservation levels of the motifs learned in the cell and histone mark models. As shown in Fig. 5a, there is positive correlation between the inference scores and the conservation levels of motifs learned in all the cell models (Tn: r = 0.15, *p* = 0.011; Tcm: r = 0.11, *p* = 0.052; Tem: r = 0.079, *p* = 0.19; and Temra: r = 0.17, *p* = 0.003), though with varying levels of significance. Moreover, as shown in Fig. 5b, there is a positive correlation between the inference scores and the conservation levels of motifs learned in the models of the four activation-related histone marks H3K4me1 (r = 0.43, *p* = 2.3e-13), H3K4me3 (r = 0.17, *p* = 0.0043), H3K27ac (r = 0.35, *p* = 2.6e-9) and H3K36me3 (r = 0.23, *p* = 0.00016). However, there is negative or no significant correlation between the inference scores and the conservation levels of motifs learned in the models of the two repression-related marks H3K9me3 (r = − 0.13, *p* = 0.036) and H3K27me3 (r = 0.063, *p* = 0.29). These results indicate that more conserved motifs learned in either the cell or histone mark models generally have higher inferences on the respective predictions than less conserved ones, with the exception that rapidly evolving motifs in the H3K9me3 mark peaks (heterochromatins) tend to have higher inferences on the prediction of cell types than more conserved ones. These observations are in line with the general understanding of the evolution of DNA sequences that functionally important sequences tend to be either more conserved due to purifying selection or evolved more rapidly due to positive selection. Thus, these results further corroborate our predicted motifs.

Interestingly, motifs learned in the Tn and Tcm models tend to be more conserved than those learned in the Tem and Temra models, and the motifs learned in the Temra model are least conserved (Fig. 5c). Thus, there is a trend that the more differentiated the cells, the less conserved the motifs learned from the corresponding models, suggesting that more conserved mechanisms might be used in the cells at the earlier stages of differentiation to specify their histone modification patterns than in the cells in the later stages of differentiation. This conclusion is consistent with the general understanding about the development of animals during embryogenesis [35]. Moreover, motifs learned in the models of gene activation-related marks H3K4me3, H3K27ac, H3K4me1 and H3K36me3 are more conserved than those learned in the models of repression-related marks H3K9me3 and H3K27me3 (Fig. 5d). This result suggests that more conserved mechanisms might be used to specify the patterns of the four activation-related marks than those used to govern the patterns of the two repression-related marks.

### The CNN models can predict cooperative TFs for specifying histone modifications in cells

**Fig. 6:**
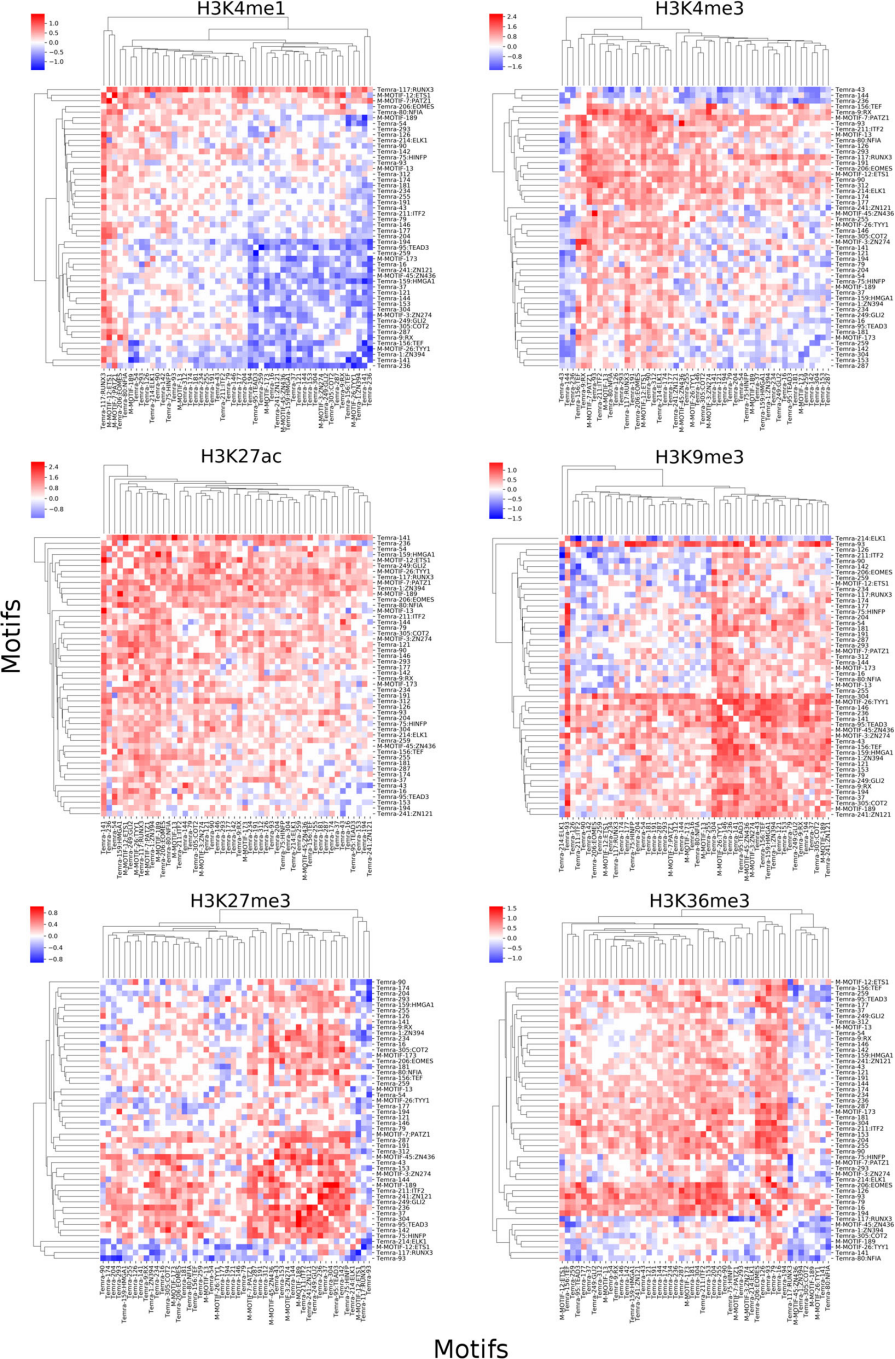
Interactions between each pair of the top 50 learned motifs on the predictions of the histone marks by the Temra cell model. The heatmaps show the values of interaction coefficient γ between the top 50 learned motifs on predicting the indicated histone marks in the Temra cell model. The scale bar shows the range of interaction coefficient γ. A negative value indicates a negative interaction while a positive value indicates a positive interaction between the pair of motifs

To see if the models can be used to identify cooperative TFs that define the histone modification patterns in the T cells, we quantified the interactions between each pair of learned motifs using a linear regression model where a positive or negative interaction coefficient indicates positive or negative interaction (Methods). To reduce the computational time, we only focused on the top 50 of learned unique motifs with the highest inference scores for both the cell models and histone mark models. Shown in Fig. 6 are the results for the Temra cell model. Clearly, there are different patterns of positive and negative interactions between the learned motifs for predicting different histone marks in the cell type. Interestingly, the motifs can be clustered into groups based on the patterns of their interactions in predicting the histone modifications. For example, in the case of predicting H3K4me1 modifications, learned motifs matching those of RUNX3, ETS1 and PATZ1 form a group with positive interactions among them; learned motifs matching those of EOMES, NFIA, ELK1, HINFP and ITF2 form a group with many putative novel motifs with largely positive interactions among them; learned motifs matching those of TEAD3, ZN121, HMGA1, ZN436, GLI1, ZN274, COT2, RX, TEF, ZN394 and TYY1 form a group with many putative novel motifs with largely negative interactions among them. Some of the predicted interactions are supported by experimental evidences. For example, we predicted ITF2 (also named T cell specific transcription factor 4 (TCF4)) had significant interactions with ETS1 for predicting histone marks H3K27ac (γ = 1.27, *p* = 3.69e-65), H3K27me3 (γ = 0.18, *p* = 0.01), H3K36me3 (γ = 0.21, *p* = 0.00077), H3K4me3 (γ = 1.15, *p* = 8.54e-57) and H3K9me3 (γ = − 0.39, *p* = 6.70e-06). In agreement with these predictions, it has been shown that ITF2 might be involved in histone acetyltransferase CBP recruitment by interacting with ETS1 [36]. Furthermore, we predicted that ITF2 had a positive interaction with RUNX3 for determining histone marks H3K27ac (γ = 1.40, *p* = 4.29e-49), H3K27me3 (γ = − 0.20, *p* = 0.013), H3K36me3 (γ = − 0.59, *p* = 6.40e-25), H3K4me1 (γ = 0.32, *p* = 8.91e-05), H3K4me3 (γ = − 1.13, *p* = 4.00e-40), and H3K9me3 (γ = − 0.18, *p* = 0.03), which is in line with the earlier finding that RUNX3 involves in regulating Wnt signaling activity by interacting with ITF2 (TCF4) in a ternary complex manner [37]. The predicted interactions between known and unknown motifs as well as between unknown motifs are likely to be novel interactions, in particular those with strong and highly significant interactions, such as the interactions for predicting the H3K27ac mark, between GLI2 and Temra 146 (γ = 2.137, *p* = 4.95e-43), between TEAD3 and Temra 54 (γ = 1.97, *p* = 4.43e-50), and between Temra 141 and Temra 146 (γ = 1.99, *p* = 4.41e-43), etc. Similar patterns of interactions were observed in the models of the other three T cell types (Additional file 1: Figures S5-S7).

Shown in Additional file 1: Figure S8. are the results for the H3K4me1 model. Again, there are distinct patterns of positive and negative interactions between the motifs for predicting different cell types by the model. As in the cases of cell models, the motifs can be clustered into groups based on the patterns of their interactions for predicting the cell types. For instance, in the case of predicting the Tn cells, the putative novel motifs M-Motif-71 and H3K4me1–30 form a group with a negative interaction; learned motifs matching those of HIC2, HXD2, TFE2, ZN547, HAND1, COT1, SMAD4, TBX1, ANDR, ZN263, THA, ZN784, ZSCA4, ZN436, PTF1A and ZN770 form a group with many putative novel motifs with largely positive interactions among them; learned motifs matching those of HXC10, PO3F3, POXJ3, HMGA2, HXC10, DLX1 and ZN250 form a group with many putative novel motifs with largely negative interactions among them. Some of the predicted interactions are supported by experimental evidences. For example, we predicted that TFE2 interacted with HAND1 for predicting Tn (γ = 5.38, *p* = 1.84e-137), Tcm (γ = 4.00, *p* = 6.94e-115), Tem (γ = 2.82, p = 1e-70) in Temra (γ = − 7.61, *p* = 1.97e-45), while it is has been reported that TFE2 (also named E47) directly interacts with HAND1 [38]. We predicted that SMAD4 interacted with ANDR for predicting Tn (γ = 2.91, *p* = 2.68e-47), Tcm (γ = 3.49, *p* = 1.86e-77), Tem (γ = 2.99, *p* = 3.47e-79) and Temra (γ = − 0.93, *p* = 0.0002), while SMAD4 is known to interact with ANDR, which might be involved in differential regulation of the androgen receptor gene transactivation [39]. We predicted that TFE2 interacted with PTF1A for predicting Tn (γ = 5.22, *p* = 3.69e-20), Tcm (γ = 3.247, *p* = 2.84e-29), Tem(γ = 2.40, p = 1.86e-13), and Temra (γ = − 5.54, *p* = 1.89e-68), while it has been reported that SMAD4 physically interacted with PTF1A and plays a crucial role in regulating signal pathways [40]. We predicted that HMGA2 interacted with SMAD4 for predicting Tn (γ = − 0.41, *p* = 0.026), Tcm (γ = − 2.24, p = 2.84e-13) and Temra (γ = 0.90, *p* = 8.77e-05), while it is known that HMGA2 interacts with SMAD3/SMAD4 to regulate SNAIL1 gene expression [41]. The predicted interactions between known and unknown motifs as well as those between unknown motifs are likely to be novel interactions, in particular those with strong and highly significant interactions, such as the negative interaction between M-Motif-71 and H3K4me1–30 for predicting Tn (γ = − 4.17, *p* = 2.75e-274), Tcm(γ = − 2.88, *p* = 3.40e-115) and Tem (γ = − 2.28, *p* = 2.36e-100), and a positive interaction for predicting Temra(γ = 3.78, *p* = 2.09e-63). Similar patterns of interactions are seen in the models of the other five histone marks (Additional file 1: Figures S9-S13).

## Discussion

DNA sequence plays a crucial role in determining its epigenomic state through interacting with the TFs and epigenome remodeling systems. However, our current understanding of these sequence determinants is still limited, and thus new methods are needed to reveal them. Recently, Whitaker and colleagues [8] trained a random forest classifier based on a set of pre-specified DNA motifs to predict six histone marks in H1 and its derived cell types with high accuracy. The results strongly support the pivotal roles of these motifs in specifying the unique epigenomes in the cells. However, this method could not discover sequence determinants ab initio, therefore, new methods are needed to gain a better understanding of the sequence determinants of epigenomes of cell types. CNNs have been proved to be a powerful approach to predict epigenomic features including TF binding [10], DNase I accessibility [13], DNA methylation [11, 42] and histone modifications [11]. And one of the advantages of CNNs, which other machine-learning methods often lack, is their ability to automatically learn the features of the objects through the filters in the convolutional layers [43]. In the case of epigenomic analysis, these features include sequence determinants that define the unique patterns of epigenetic modifications in different cell types produced during embryogenesis and development. Thus, CNNs can be a powerful approach to real the epigenomic sequence determinants.

Indeed, efforts have been made to interpret the sequence features learned by CNN models for predicting epigenomic marks [10–13]. However, these studies used a single mixed model to predict a combination of multiple epigenetic marks with multiple cell types, thus lack the power of comparative analyses for the learned sequence features. To overcome this limitation and facilitate interpreting CNN models which can be otherwise highly challenging [44], we developed two types of CNN models to capture the sequence features for various histone modifications in different cell types: 1) the cell type model for predicting patterns of various histone modifications in a cell type, and 2) the histone mark model for predicting various cell types based on a histone mark. In this way, by comparing the motifs earned in different cell type models, we could identify the common and unique motifs that specify unique patterns of various histone modifications in a cell type; and by comparing the motifs learned in different histone mark models, we could detect the common and unique motifs that determine different patterns of the same histone mark in different cell types. Furthermore, the models enable us to evaluate the inferences of learned motifs and their interactions on the prediction accuracy, thereby predicting roles of each motif in specifying the epigenome and the type of cells.

To validate this strategy, we applied it to a dataset of six histone marks derived from four well-studied CD_4_^+^ T cell types in humans, i.e., Tn, Tcm, Tem and Temra. Both our histone mark models and cell type models achieved very high accuracy and were highly robust when tested on the dataset for H1 and its derived cell types, suggesting that our models have largely learned the relevant sequence features in determining the unique histone mark patterns in these cells. Not surprisingly, a large portion of the learned motifs in the first convolutional layers in the models resemble those of TFs that are known to play crucial roles in T cell development, while the remaining ones could be novel motifs of unknown TFs participating in T cell differentiation. By comparing the motifs learned in different cell models, we predicted that the unique patterns of various histone modifications in each cell type were largely determined by a unique set of motifs (Fig. 2b and c) and at the same time, the number of common motifs shared by two cell models reflected the linear lineage relationships of the four CD4+ T cell types (Fig. 2g), which is consistent with the results based on DNA methylation, DNase hypersensitivity and transcription patterns in the earlier study that produced the datasets used in our analysis. Furthermore, by comparing the motifs learned in different histone mark models, we predicted that different patterns of the same histone marks in different cell types were largely determined by a unique set of motifs (Fig. 2b and c), while the number of common motifs shared by two histone mark models reflected their co-modification and exclusiveness natures (Fig. 2h). All these results suggest that at least most of the learned motifs are likely to be authentic and play roles in T cell differentiation. Moreover, by computing the inference scores of the learned motifs, we further predicted the specific roles of each learned motif in determining the patterns of various histone modifications in a cell (Fig. 3a and c), or different patterns of the same histone modification in different cells (Fig. 3b and d). Finally, by computing an interaction score, we predicted the interactions of the cognate TFs of the learned motifs in either the cell models or histone mark models. Some of these predictions have experimental supports. Thus, our results support the hypothesis that sequences ultimately determine the unique epigenomes of different cell types through their interactions with TFs, epigenome remodeling system and extracellular cues during cell differentiation in a stepwise manner. Therefore, the motifs learned in our CNN models are highly interpretable and may provide insights into the underlying molecular mechanisms of establishing the unique histone modifications in different cell types.

## Conclusions

We have developed two types of highly accurate CNNs constructed for cell types and for histone marks to predict the different histone marks in a cell type and different patterns of same mark in different cells, respectively. We showed that both the unique histone modification patterns in a cell type and the different patterns of the same histone mark in different cell types are determined by a set of motifs with unique combinations. The level of sharing motifs learned in the different cell models reflects the lineage relationships of the cells, while the level of sharing motifs learned in different histone mark models reflects their functional relationships. The models enable the prediction of the importance of the learned motifs and their interactions in determining specific histone mark patterns in the cell types. Therefore, the motifs learned in the models are highly interpretable and may provide insights into the underlying molecular mechanisms of establishing the unique histone modifications in different cell types. Our results suggest the hypothesis that DNA sequences ultimately determine the unique epigenomes of different cell types through their interactions with TFs, epigenome remodeling system and extracellular cues during cell differentiation in a stepwise manner.

## Methods

### The datasets

#### Human CD4+ T cells dataset

We downloaded from European Genome-Phenome Archive the ChIP-seq datasets for six histone marks H3K4me1, H3K4me3, H3K27me3, H3K27ac, H3K9me3 and H3K36me3 in four different human CD_4_^+^ T cell types native T (Tn), central memory T (Tcm), T effector memory (Tem), and CD_4_^+^ terminally differentiated CD_45_RA^+^ memory T (Temra) cells [14].

#### Human embryonic stem cells dataset

We downloaded from the Roadmap Epigenomics Project [16] the ChIP-seq datasets for six histone marks H3K4me1, H3K4me3, H3K27me3, H3K27ac, H3K9me3 and H3K36me3 in H1 human embryonic stem cells (H1) and in four cell types derived from H1, including trophoblast-like (TBL), mesendoderm (ME), mesenchymal (MSC) and neural progenitor (NPC) cells.

### Peak calling, filtering and merging

To identify genome regions that are modified by different histone marks, we called tight and broad histone modification peaks [8] using MACS2 [45]. The tight peaks including H3K27ac, H3K4me1 and H3K4me3 are typically < 1 kbp. The broad peaks including H3K27me3, H3K36me3 and H3K9me3 are typically > 1 kbp. The tight peaks were called as follows:

macs2 callpeak -t bam/tagAlign file -n name -c control file –outdir output dir -g hs -q 0.05 –nomodel –extsize fragment length.

The broad peaks were called as follows:

macs2 callpeak -t bam/tagAlign file -n name -c control file –outdir output dir -g hs –broad –broad-cutoff 0.1 –nomodel –extsize fragment size.

The fragment sizes were estimated using phantompeakqualtools [46, 47].

We discarded peaks whose − *log* 10(*qvalue*) was less than 2 or whose length was greater than 10,000 bp for their low quality or too long length. We also removed the peaks that overlapped the blacklisted regions of the human genome [48], which are regions showing artificially high signal in all NGS experiments. To ensure only regions of high confidence were considered, we only used the intersection of at least two replicates when possible. We extracted and merged the peaks using BedTools [49], and used the CRCh37/hg19 genome assembly for all the analyses.

### Data representation

To prepare the input for the deep CNN models, we segmented the human whole genome (CRCh37/hg19) into 200-bp bins [11]. For a cell model, we labeled each bin with a binary vector with each bit indicating whether it was modified by the corresponding histone mark (1) or not (0) in the cell type. For a histone mark model, we labeled each bin with a binary vector with each bit indicating whether it was modified by the mark in the corresponding cell type (1) or not (0). We say that a bin overlaps with a peak if the overlapping portion of the bin with the peak is above a threshold. To achieve the best prediction results, we tested different thresholds of 0.5, 0.6, 0.7, 0.8 and 0.9, and chose the threshold with the highest accuracy for the final analysis. We discarded the bins that had no overlap with any histone modifications. We then extended the 200-bp bin into 1000-bp sequence centered on the middle of the 200-bp bin for context learning [11]. Each extended 1000-bp sequence was represented by a 1000 × 4 binary matrix as the input to the CNN models, and each row was one hot vector to represent the presence or absence of A, C, G, T at the nucleotide position. If a nucleotide position is N in the genome, we represented it as [0.25, 0.25, 0.25, 0.25] [13].

### Convolutional neural networks

**Fig. 7:**
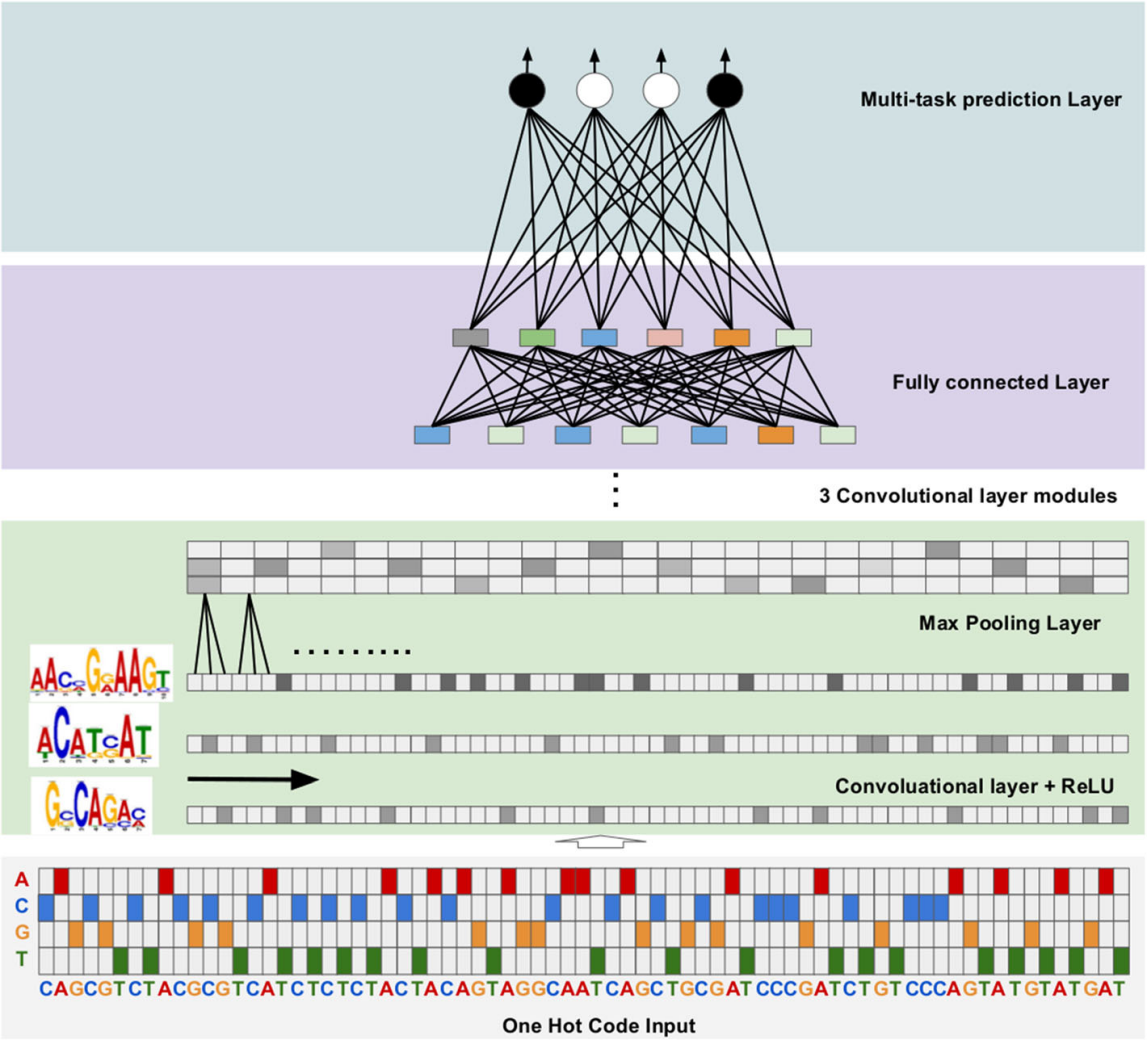
An Illustrative schematic of the convolutional neural network models. ReLU is the rectified linear unit activation function, for details, see the text

CNNs are a type of feed-forward artificial neural networks, usually consisting of an input layer, multiple convolutional layers, one or more fully connected layers and an output layer. Our CNN models (Fig. 7) are made of a stack of three units each consisting of a convolutional layer, a pooling layer and a batch normalization layer, followed by a fully connected layer and an output layer. We apply a rectified linear unit (ReLU) transform as the activation function after a convolution layer (Fig. 7), which helps to prevent vanishing gradient problem [50, 51]:

1 $$ Convolution{(X)}_{lk}= ReLU\left(\sum \limits_{l=0}^{L-1}\sum \limits_{d=0}^{D-1}{W}_{ld}^k{X}_{i+l,d}\right), $$

where X is the input, L is the input length, D is the input dimension, i is the output position, and k is filters’ index.

2 $$ ReLU(x)=\max \left(0,x\right) $$

To decease internal covariate shift and accelerate training, we apply a batch normalization layer after the convolutional layer [52]. Furthermore, we apply a max pooling layer after the batch normalization layer, which extracts the maximum activation value from each receptive field in the prior layer. Three convolutional layers contain 320, 300 and 300 kernels, respectively, and the fully connected layer has 1000 units with a sigmoid activation function feeding into the output layer (Fig. 7). We use a sigmoid function as the activation function of the output layer to conduct multi-task prediction,

3 $$ y(X)= Sigmoid(X)=\frac{1}{1+{e}^{- WX}} $$

where y(X) is the prediction of the output layer, X is the output of the previous layer, W is the weight matrix of the output layer. We implemented the CNN models using Theano [53] and Lasagne [54].

### Model training, validation and evaluation

We split a dataset into a training dataset, a validation dataset and a test dataset with a ratio about 2:1:1, and the objective function is binary cross entropy. We apply a stochastic gradient descent to minimize the objective function by updating all model parameters using RMSprop with a learning rate 0.001 on minibatch [55]. To avoid overfitting, we apply L1 and L2 regularization terms and the early stopping strategy. To keep the filters free to grow based on input sequences, we only apply L1 and L2 regularization terms to the fully connected layer. To quickly choose the best set of hyperparameters of the models, we use parallel random search and apply L1 and L2 as well as maximum epochs as shown in Table 1.

**Table 1:** Hyper-parameter configurations for training the models

Trail	L1	L2	Patience	Max epochs	Batch size
1	1e-07	2e-08	5000	20	128
2	2e-07	4e-08	5000	20	128
3	3e-07	8e-08	5000	20	128
4	4e-07	2e-07	5000	20	128
5	5e-07	4e-07	5000	20	128
6	6e-07	8e-07	5000	20	128

Overlap is 0.5, 0.6, 0.7, 0.8, 0.9

We performed the receiver operating characteristic (ROC) curve analysis and used the area under the curve (AUC) to evaluate the performance of the models. We also define the accuracy of a model as follows:

4 $$ Accuracy=\frac{TP+ TN}{TP+ FN+ TN+ FP} $$

where TP is true positive, TN true negative, FN false negative and FP false positive.

### Interpretation of the kernels/filters in the first convolutional layer

The first convolutional layer of the models scans the DNA sequences with its kernel or filters to capture the k-mer motifs that differentiate modified and unmodified DNA sequences. Thus these filters potentially correspond to the binding motifs of TFs or chromatin remodeling proteins whose interactions with the motifs may lead to the specific modifications at the loci. To reveal such these motifs, we construct a position weight matrices (PWMs) for each filter by extracting k-mers in the test dataset, which has a score against the filter greater than a threshold defined as,

5 $$ Threshold=\left({\alpha}_{max}-{\alpha}_{min}\right)\times \beta, $$

where *α*_*max*_ and *α*_*min*_ are the maximum and minimum activations for a k-mer across all sequences in the test dataset, respectively, and β is a ratio constant. For each filter, we evaluated β ranging from 0.3 to 0.8, and chose the resulting PWM with the highest information content. We discard the resulting PWMs with 0 information content. To evaluate the inference of a filter on the model’s prediction, we nullify forward information of the filter by setting its output as its mean output over all nucleotides of all sequences in the test dataset [13], and quantify each filter’s inference as sum of square of the difference of the prediction probability in the test dataset before and after the nullification as follows,

6 $$ Influence(k)=\sum \limits_{x\in D}{\left({P}_{pre}(x)-{P}_{aft}(x)\right)}^2, $$

where D is the test dataset and *P*_*pre*_(*x*) and *P*_*aft*_(*x*) are the prediction probabilities before and after nullifying the filter k, respectively.

### Motif conservation analysis

We used Fimo [56] to scan sequences for binding sites of each motif as follows:

fimo –parse-genomic-coord –thresh 1e-5 –bgfile fasta file background model –oc output_folder motifs_meme target_sequences.

We used a 5th-order Markov model [57] to generate the background sequences as follows:

fasta-get-markov -m 5 -dna sequences background_model.

We extracted the phastCons [58] score for each position in each binding site, and calculated a conservation score for each motif as the mean the PhastCons scores of all the binding sites of each motif learned in the models. To study the relationship between the inferences of the learned motifs and their conservation levels, we computed the Pearson correlation coefficient between them, and tested the null hypothesis of non-correlation using two-tailed *p*-values,

7 $$ r=\frac{\mathit{\operatorname{cov}}\left(I,C\right)}{\sqrt{\mathit{\operatorname{var}}(I)}\sqrt{\mathit{\operatorname{var}}(C)}}, $$

where I, C are the inference and phastCons scores of motifs, respectively, and r Pearson correlation coefficient.

### Merging highly similar motifs

To merge similar motifs learned in all the cell and histone mark models, we compared each motif with all other motifs using TOMTOM [59], and constructed a graph by connecting two motifs if they were a pair of bidirectional best hits with a minimum overlap of 7 bps and E value < 0.1. We then cut the network into connected components using Networkx [60]. Some components are singletons containing a single original motif, while others are formed by multiple highly similar original motifs. We consider each of these components as a unique motif. To find the PWM for the merged motifs, we performed motifs finding on the merged binding sites using ProSampler [61].

### Prediction of interactions between cognate TFs of learned motifs

To predict possible interactions between the cognate TFs of the learned motifs, we applied a linear model to the changes in the prediction probability for random selected 2000 sequences after the two motifs were simultaneously nullified, defined as:

8 $$ \Delta {P}_{ij}=\alpha \times \Delta {P}_i+\beta \times \Delta {P}_j+\gamma \times \Delta {P}_i{P}_j, $$

where Δ*P*_*ij*_ is the sum square of changes in the prediction probability after simultaneously nullifying motifs i and j, *∆P*_*i*_ and *∆P*_*j*_ are the sum square of changes in the prediction probabilities after nullifying motifs i and j, respectively, and *α*, *β* and *γ* are constants. Clearly the absolute value of *γ* reflects the intensity of the interaction, while its sign (+/−) indicates a positive or negative interaction. Therefore, we call *γ* the interaction coefficient and used it to quantify the interaction between two motifs.

## Additional files

Additional file 1: **Figure S1.** Performance of the CNN models of the five cell types for predicting the six histone marks. **Figure S2.** Performance of the CNN models of the six histone marks for predicting the four cell types. **Figure S3.** Performance of the CNN models of the six histone marks for predicting the five cell types. **Figure S4.** Influences of the learned motifs on the prediction of each cell type by the histone mark models. **Figure S5.** Interactions between each pair of top 50 learned motifs on the prediction of the six marks by the Tn cell model. **Figure S6.** Interactions between each pair of top 50 learned motifs on the prediction of the six marks by the Tcm cell model. **Figure S7.** Interactions between each pair of top 50 learned motifs on the prediction of the six marks by the Tem cell model. **Figure S8.** Interactions between each pair of the top 50 learned motifs on the prediction of the four cell types by the H3K4me1 model. **Figure S9.** Interactions between each pair of top 50 learned motifs on the prediction of the four cell types by the H3K4me3 model. **Figure S10.** Interactions between each pair of top 50 learned motifs on the prediction of the four cell types by the H3K9me3 model. **Figure S11.** Interactions between each pair of top 50 learned motifs on the prediction of the four cell types by the H3K27ac model. **Figure S12.** Interactions between each pair of top 50 learned motifs on the prediction of the four cell types by the H3K27me3 model. **Figure S13.** Interactions between each pair of top 50 learned motifs on the prediction of the four cell types by the H3K36me3 model. (DOCX 10608 kb)

## Data Availability

Human embryonic stem cells dataset analyzed during the current study are available in the NIH Roadmap Epigenomics Mapping Consortium repository, https://egg2.wustl.edu/roadmap/data/byFileType/alignments/consolidated/ . Human CD4+ T cells dataset analyzed during the current study are available in The German epigenome programme ‘DEEP’ repository, http://deep.dkfz.de/#/experiments .

## Abbreviations

AUC: Area under the ROC curve
CNNs: Convolutional neural networks
FN: False negative
FP: False positive
H1: H1 human embryonic stem cells
ME: Mesendoderm cells
M-motif: Merged Motif
MSC: Mesenchymal cells
NPC: Neural progenitor cells
PWM: Position weight matrix
ReLU: Rectified linear unit
ROC: Receiver operating characteristic (ROC)
TBL: Trophoblast-like cells
TCF4: T cell specific transcription factor 4
Tcm: Central memory T cells
Tem: T effector memory cells
Temra: Terminally differentiated CD_45_RA^+^ memory
TF: Transcription factor
Tn: Native T cells
TN: True negative
TP: True positive
