# Supplementary material for: Deciphering epigenomic code for cell differentiation using deep learning
Source: BMC Genomics. 2019 Sep 12;20:709. doi: 10.1186/s12864-019-6072-8 (PMC6739944; doi:10.1186/s12864-019-6072-8)
Supplement: Supplementary file 1 — Figure S1. Performance of the CNN models of the five cell types for predicting the six histone marks. Figure S2. Performance of the CNN models of the six histone marks for predicting the four cell types. Figure S3. Performance of the CNN models of the six histone marks for predicting the five cell types. Figure S4. Influences of the learned motifs on the prediction of each cell type by the histone mark models. Figure S5. Interactions between each pair of top 50 learned motifs on the prediction of the six marks by the Tn cell model. Figure S6. Interactions between each pair of top 50 learned motifs on the prediction of the six marks by the Tcm cell model. Figure S7. Interactions between each pair of top 50 learned motifs on the prediction of the six marks by the Tem cell model. Figure S8. Interactions between each pair of the top 50 learned motifs on the prediction of the four cell types by the H3K4me1 model. Figure S9. Interactions between each pair of top 50 learned motifs on the prediction of the four cell types by the H3K4me3 model. Figure S10. Interactions between each pair of top 50 learned motifs on the prediction of the four cell types by the H3K9me3 model. Figure S11. Interactions between each pair of top 50 learned motifs on the prediction of the four cell types by the H3K27ac model. Figure S12. Interactions between each pair of top 50 learned motifs on the prediction of the four cell types by the H3K27me3 model. Figure S13. Interactions between each pair of top 50 learned motifs on the prediction of the four cell types by the H3K36me3 model. (DOCX 10608 kb) [file 12864_2019_6072_MOESM1_ESM.docx]

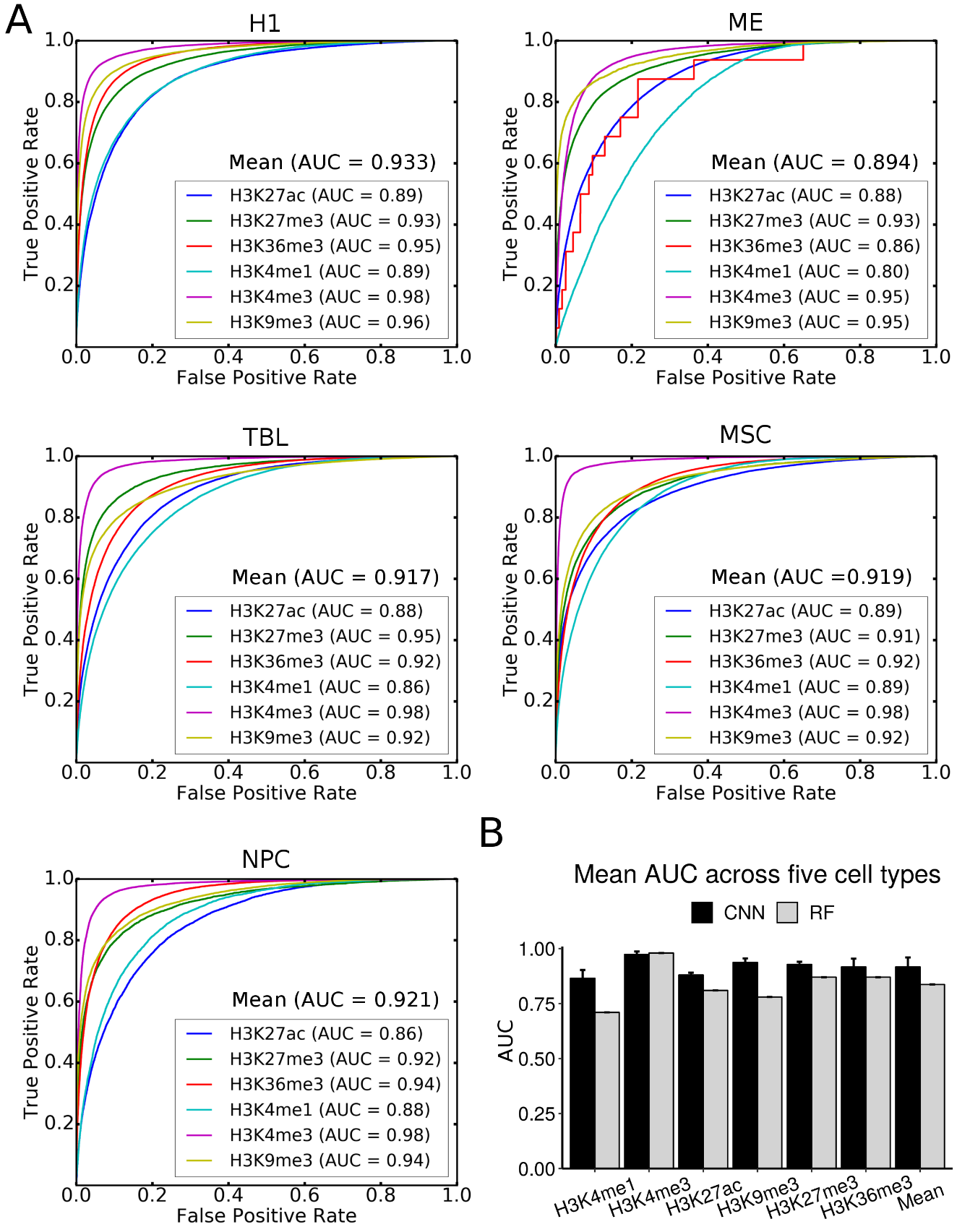


**Figure S1. Performance of the CNN models of the five cell types for predicting the six histone marks. A.** The ROCs of the H1, ME, TBL, MSE and NPC models for predicting the six histone marks. **B.** Average AUCs achieved by our CNN models and those obtained by the random forest-based models for the marks across the five cell type models. The error bars for the random forest-based models are not shown due to their unavailability.


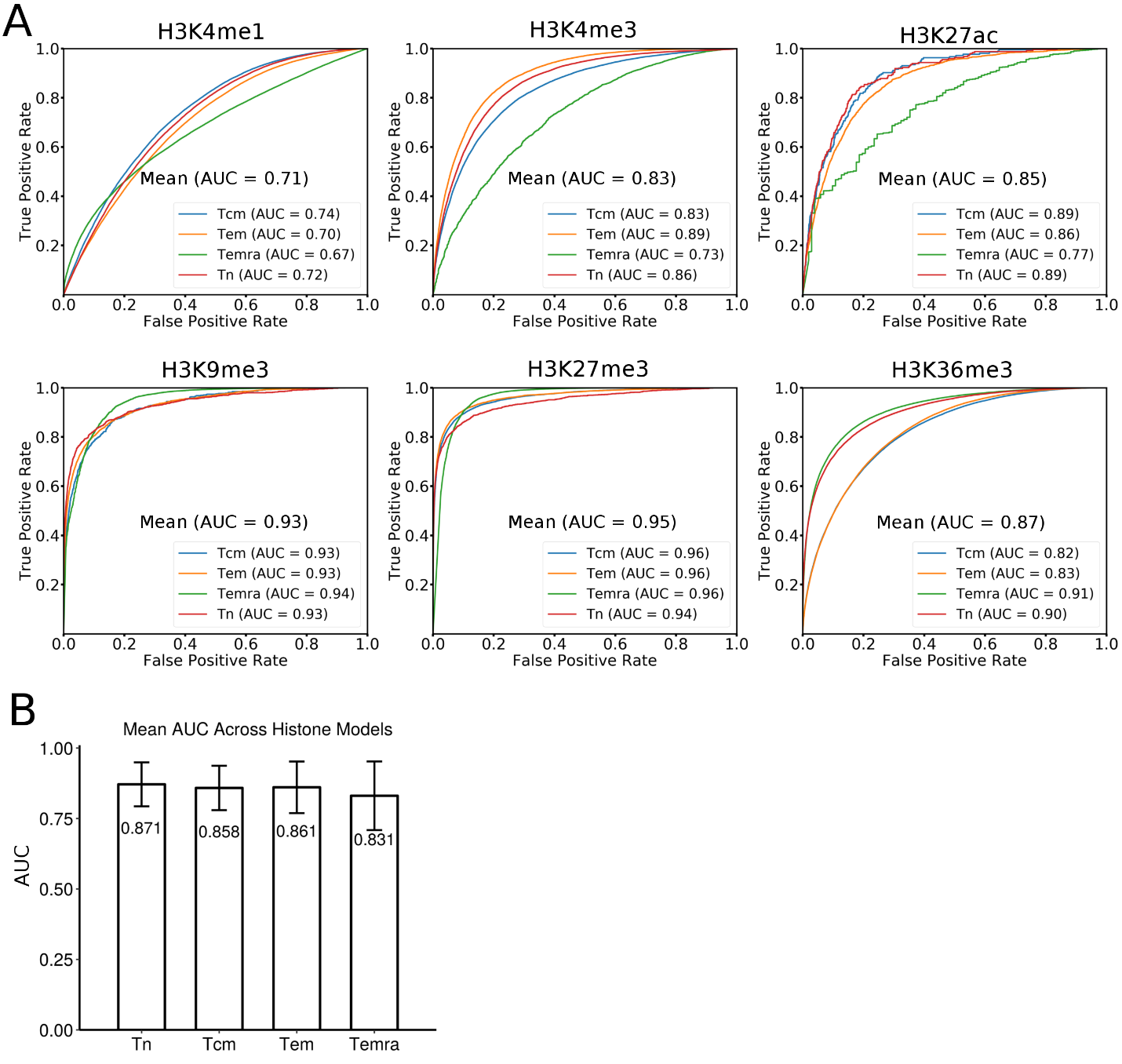


**Figure S2.** **Performance of the CNN models of the six histone marks for predicting the four cell types. A.** ROCs of the H3K4me1, H3K4me3, H3K9me3, H3K27ac, H3K27me3 and H3K36me3 models for predicting the four cell types. **B.** Mean AUC for each cell type model across the six histone mark models.


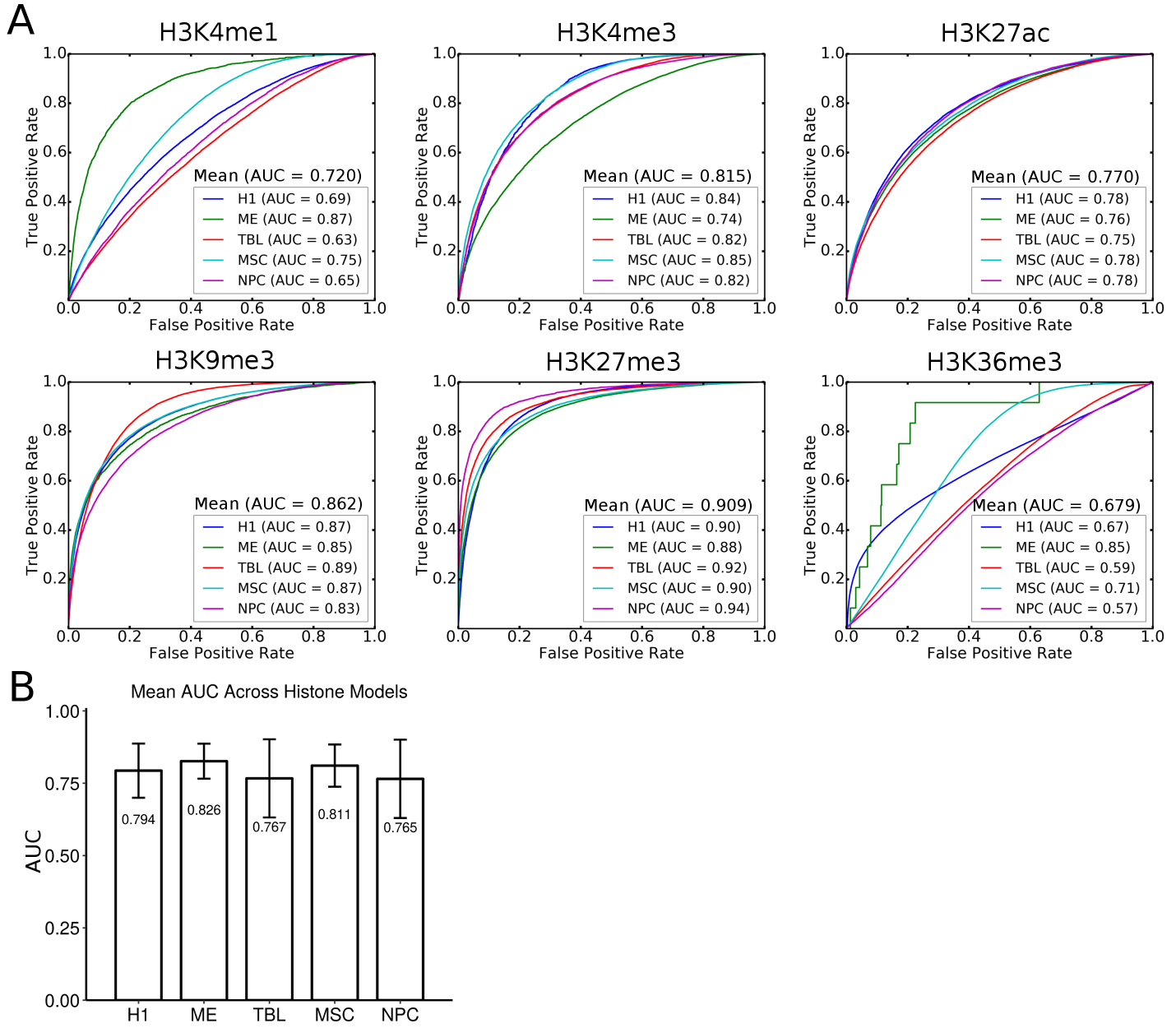


**Figure S3. Performance of the CNN models of the six histone marks for predicting the five cell types. A.** ROCs of the H3K4me1, H3K4me3, H3K9me3, H3K27ac, H3K27me3 and H3K36me3 models for predicting the five cell types. **B.** Mean AUC for each cell type across the six histone mark models.


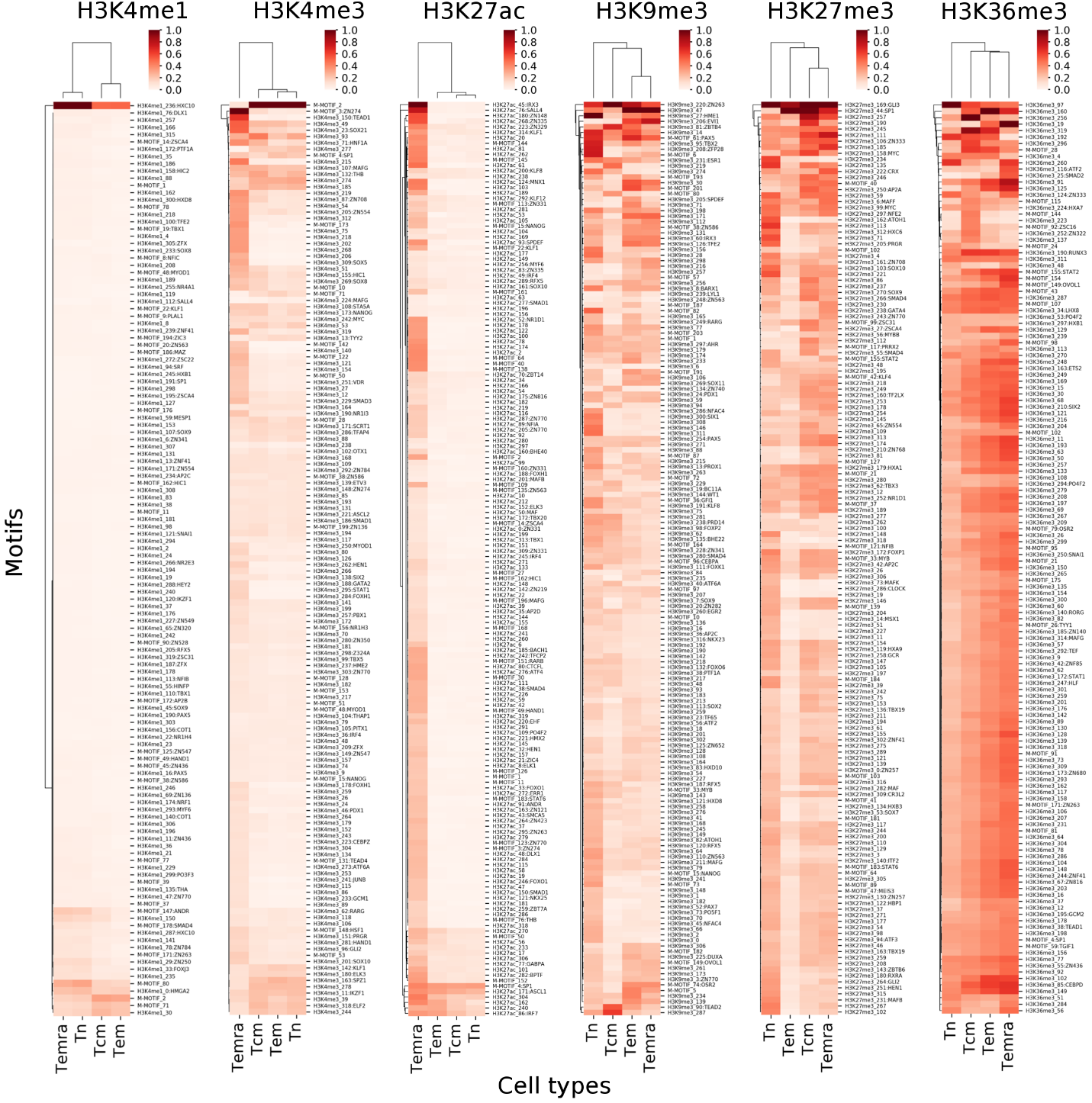


**Figure S4. Influences of the learned motifs on the prediction of each cell type by the histone mark models.** The heatmaps show the influence scores of the top 100 learned motifs on predicting the four cell types in the indicated histone mark models. The scale bar shows range of the inference score of a motif on a cell type.


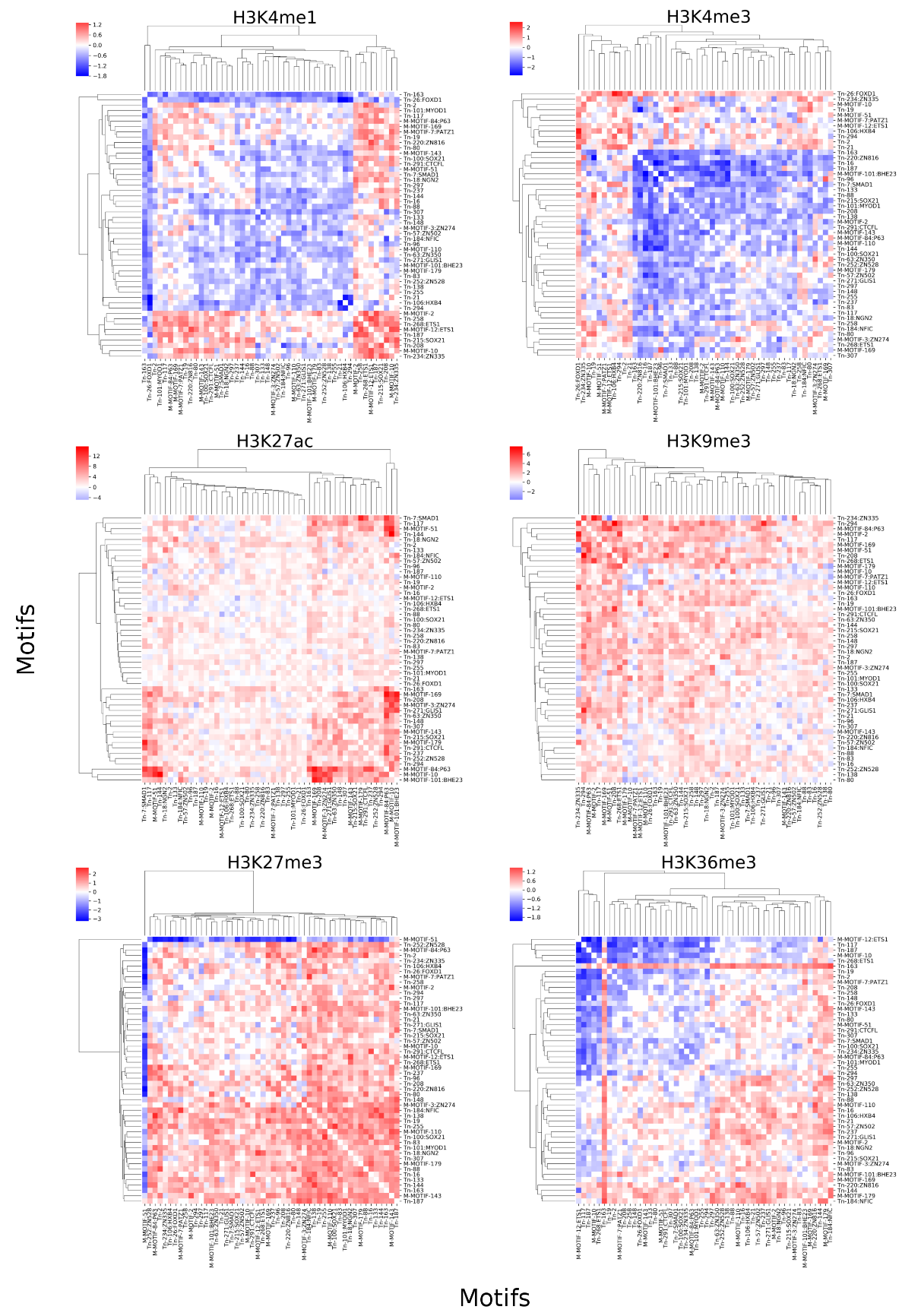


**Figure S5. Interactions between each pair of top 50 learned motifs on the prediction of the six marks by the Tn cell model.** The heatmaps show the values of interaction coefficient γ between the top 50 learned motifs on predicting the indicated histone marks in the Tn cell model. The scale bar shows range of interaction coefficient γ. A negative value indicates a negative interaction while a positive value indicates a positive interaction between the pair of motifs.


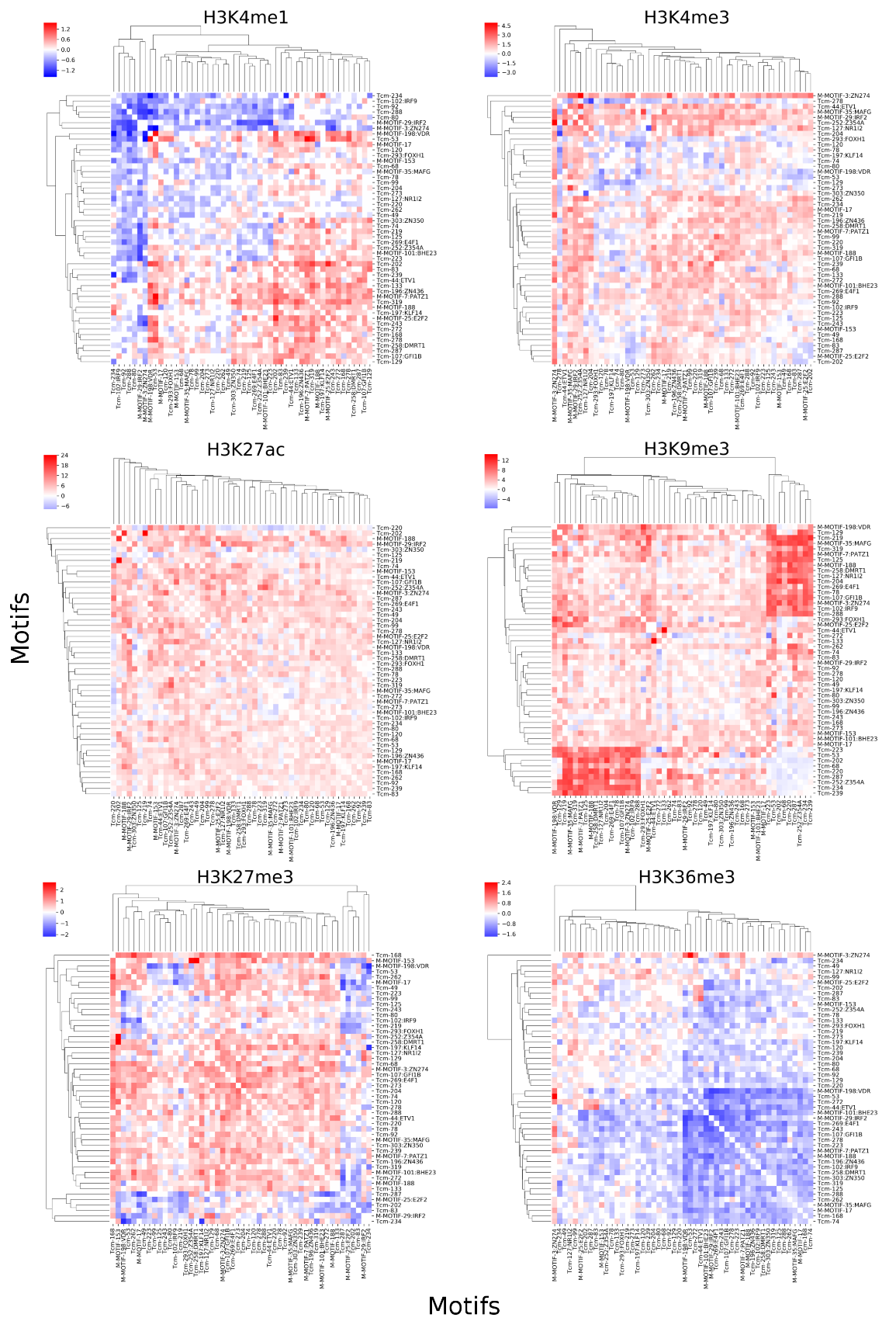


**Figure S6. Interactions between each pair of top 50 learned motifs on the prediction of the six marks by the Tcm cell model.** The heatmaps show the values of interaction coefficient γ between the top 50 learned motifs on predicting the indicated histone marks in the Tcm cell model. The scale bar shows range of interaction coefficient γ. A negative value indicates a negative interaction while a positive value indicates a positive interaction between the pair of motifs.


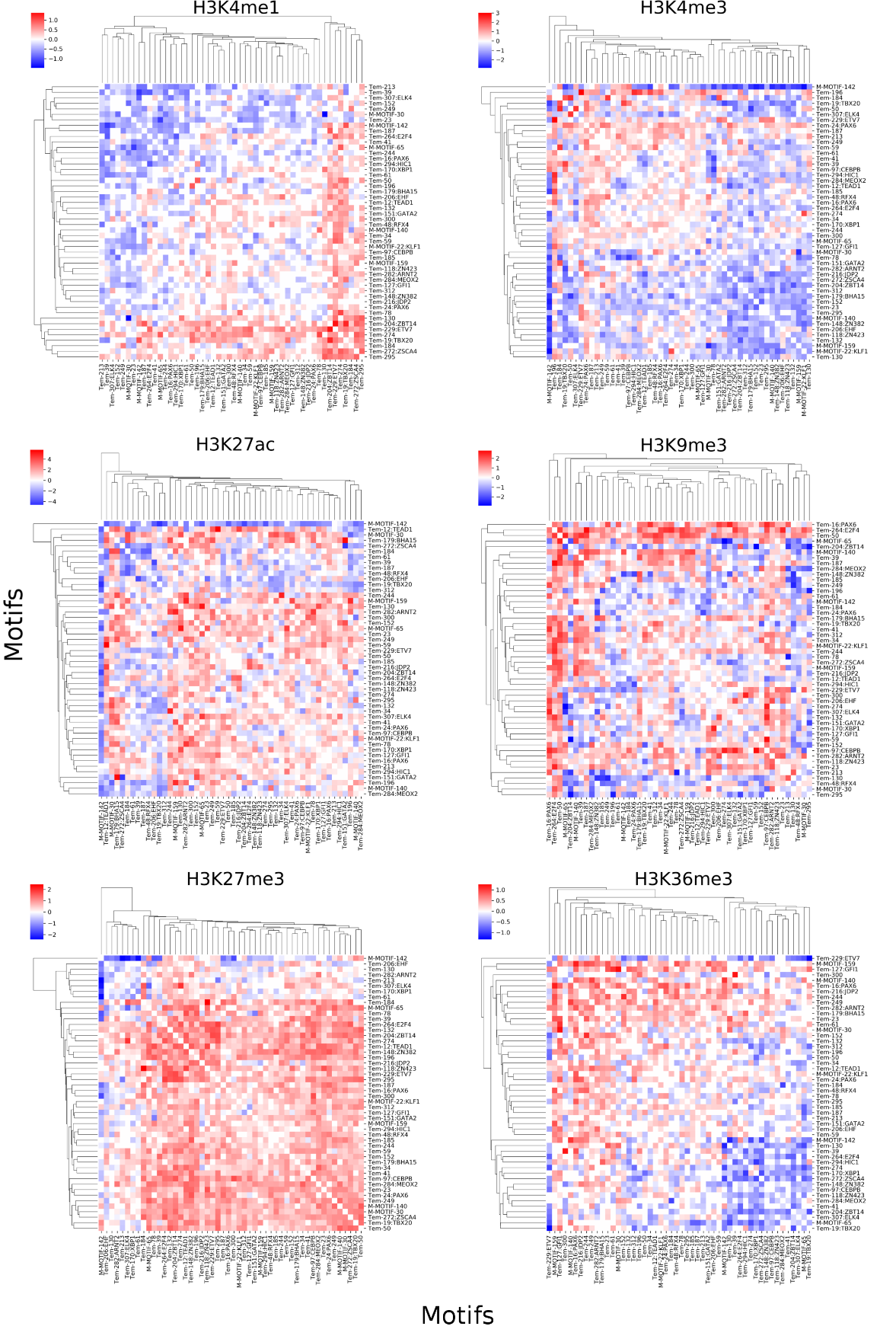


**Figure S7. Interactions between each pair of top 50 learned motifs on the prediction of the six marks by the Tem cell model.** The heatmaps show the values of interaction coefficient γ between the top 50 learned motifs on predicting the indicated histone marks in the Tem cell model. The scale bar shows range of interaction coefficient γ. A negative value indicates a negative interaction while a positive value indicates a positive interaction between the pair of motifs.

.

*
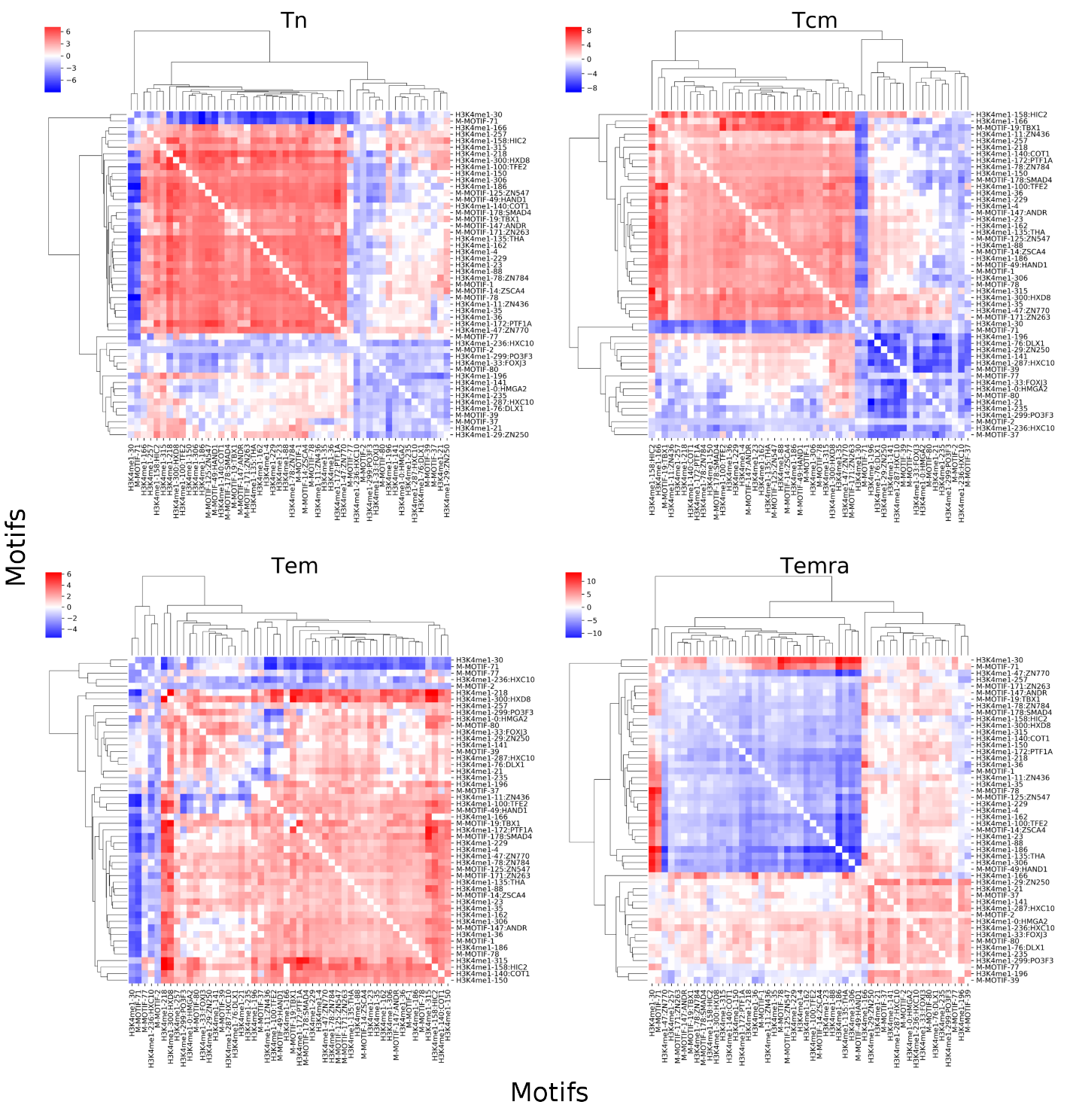
*

**Figure S8. Interactions between each pair of the top 50 learned motifs on the prediction of the four cell types by the H3K4me1 model.** The heatmaps show the values of interaction coefficient γ between the top 50 learned motifs on predicting the indicated cell types in the H3K4m1 model. The scale bar shows range of interaction coefficient γ. A negative value indicates a negative interaction while a positive value indicates a positive interaction between the pair of motifs.

­


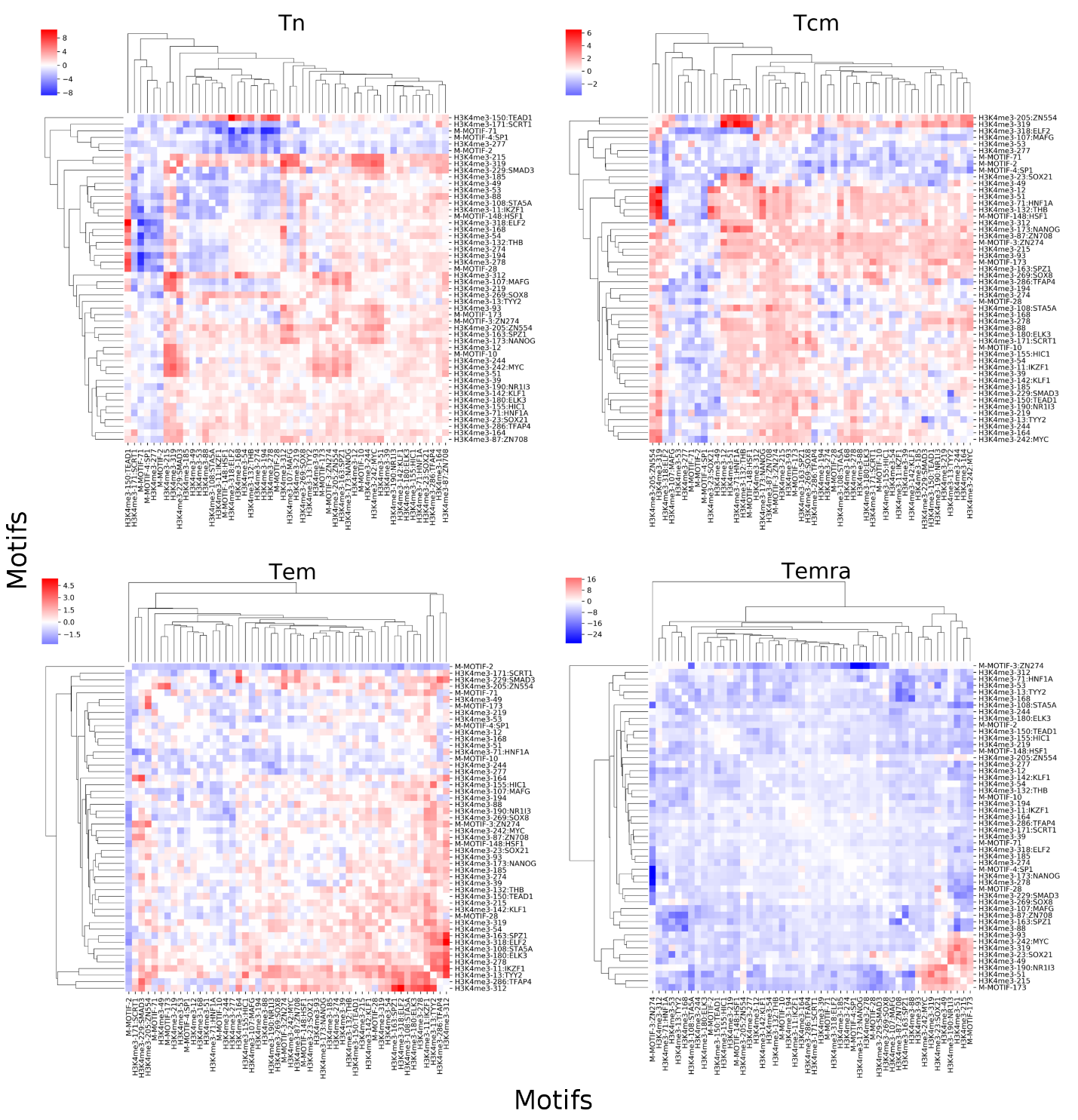


**Figure S9. Interactions between each pair of top 50 learned motifs on the prediction of the four cell types by the H3K4me3 model.** The heatmaps show the values of interaction coefficient γ between the top 50 learned motifs on predicting the indicated cell types in the H3K4me3 model. The scale bar shows range of interaction coefficient γ. A negative value indicates a negative interaction while a positive value indicates a positive interaction between the pair of motifs.


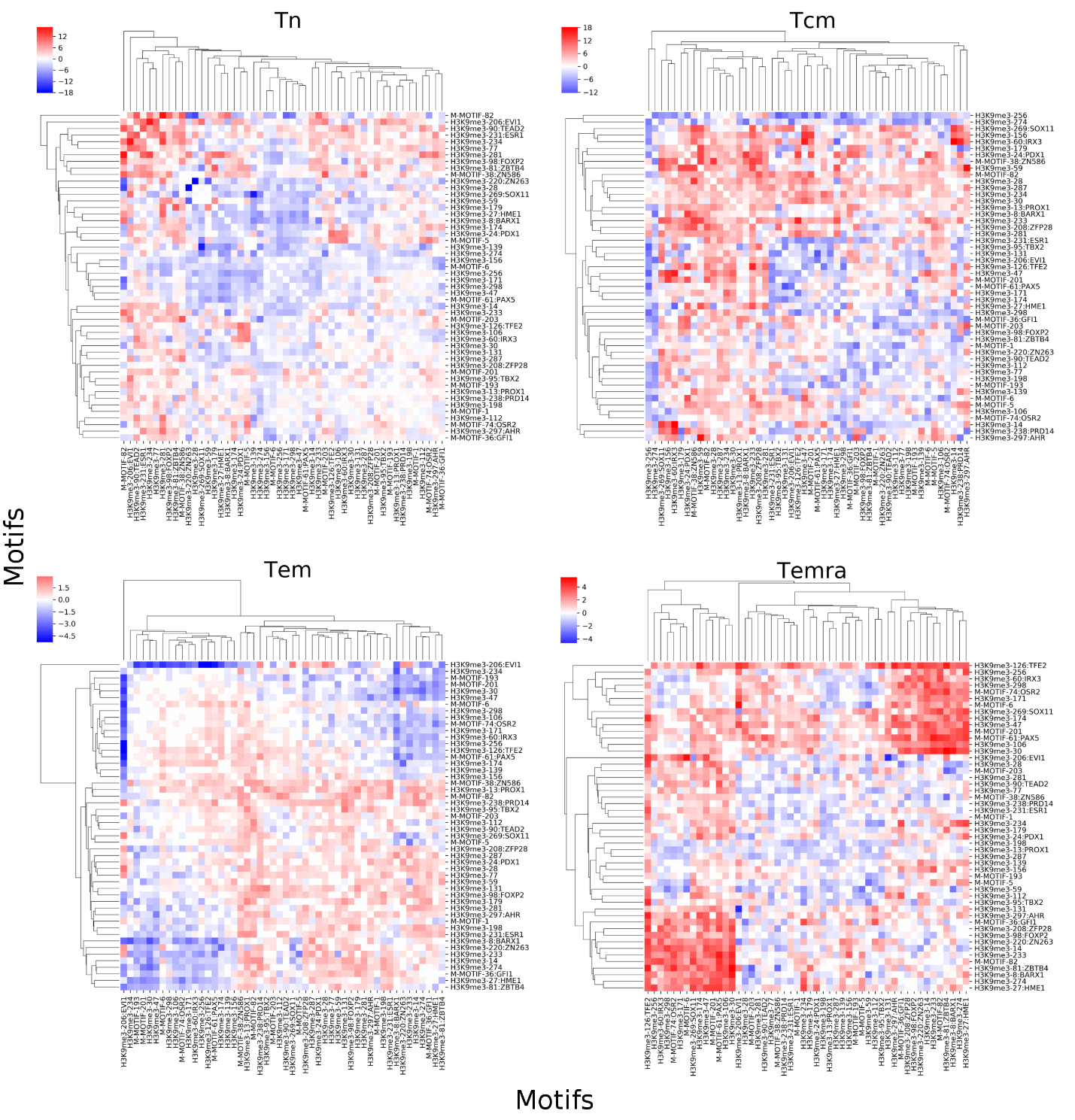


**Figure S10. Interactions between each pair of top 50 learned motifs on the prediction of the four cell types by the H3K9me3 model.** The heatmaps show the values of interaction coefficient γ between the top 50 learned motifs on predicting the indicated cell types in the H3K9me3 model. The scale bar shows range of interaction coefficient γ. A negative value indicates a negative interaction while a positive value indicates a positive interaction between the pair of motifs.


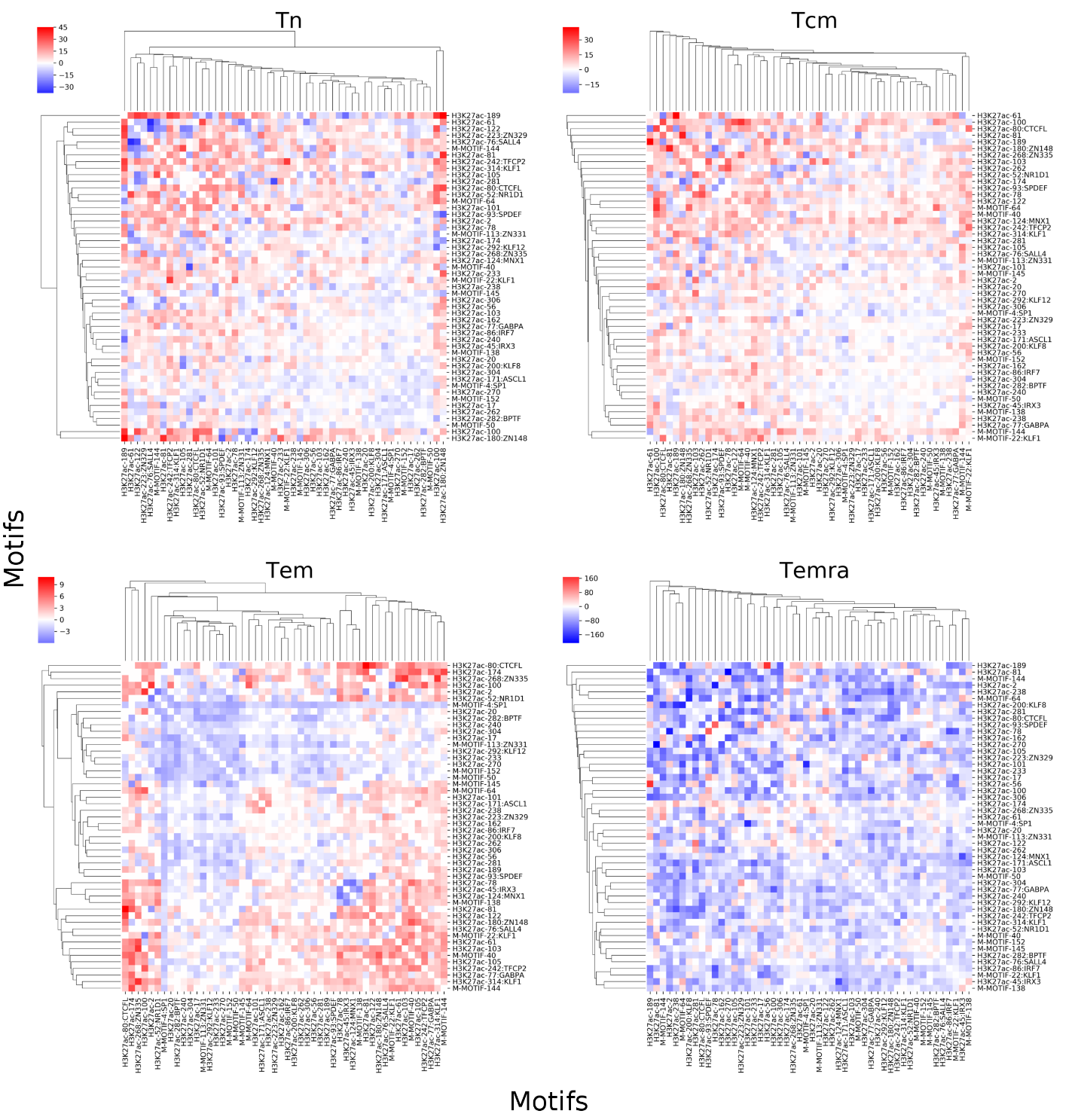


**Figure S11. Interactions between each pair of top 50 learned motifs on the prediction of the four cell types by the H3K27ac model.** The heatmaps show the values of interaction coefficient γ between the top 50 learned motifs on predicting the indicated cell types in the H3K27ac model. The scale bar shows range of interaction coefficient γ. A negative value indicates a negative interaction while a positive value indicates a positive interaction between the pair of motifs.


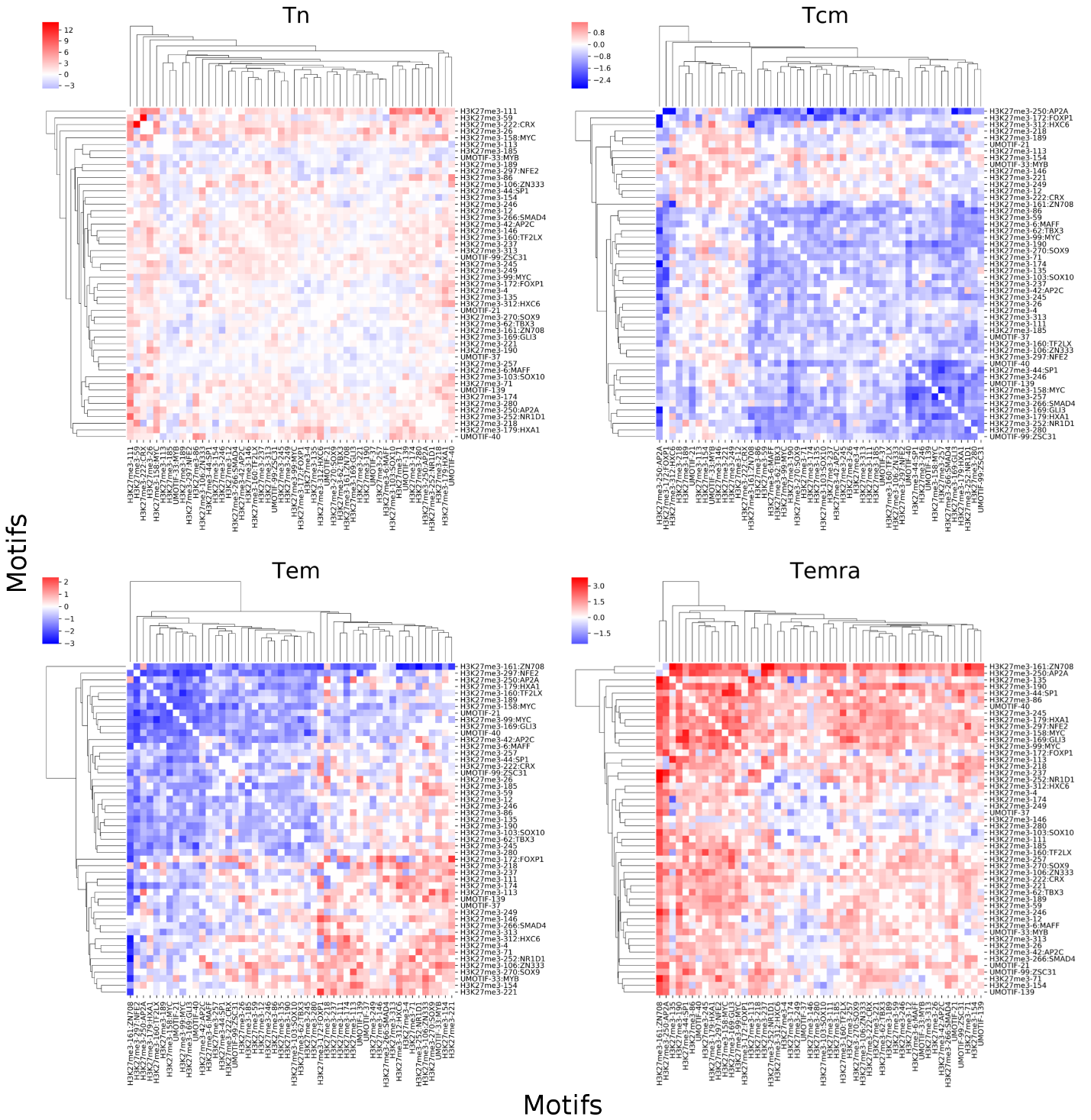


**Figure S12. Interactions between each pair of top 50 learned motifs on the prediction of the four cell types by the H3K27me3 model.** The heatmaps show the values of interaction coefficient γ between the top 50 learned motifs on predicting the indicated cell types in the H3K27me3 model. The scale bar shows range of interaction coefficient γ. A negative value indicates a negative interaction while a positive value indicates a positive interaction between the pair of motifs.


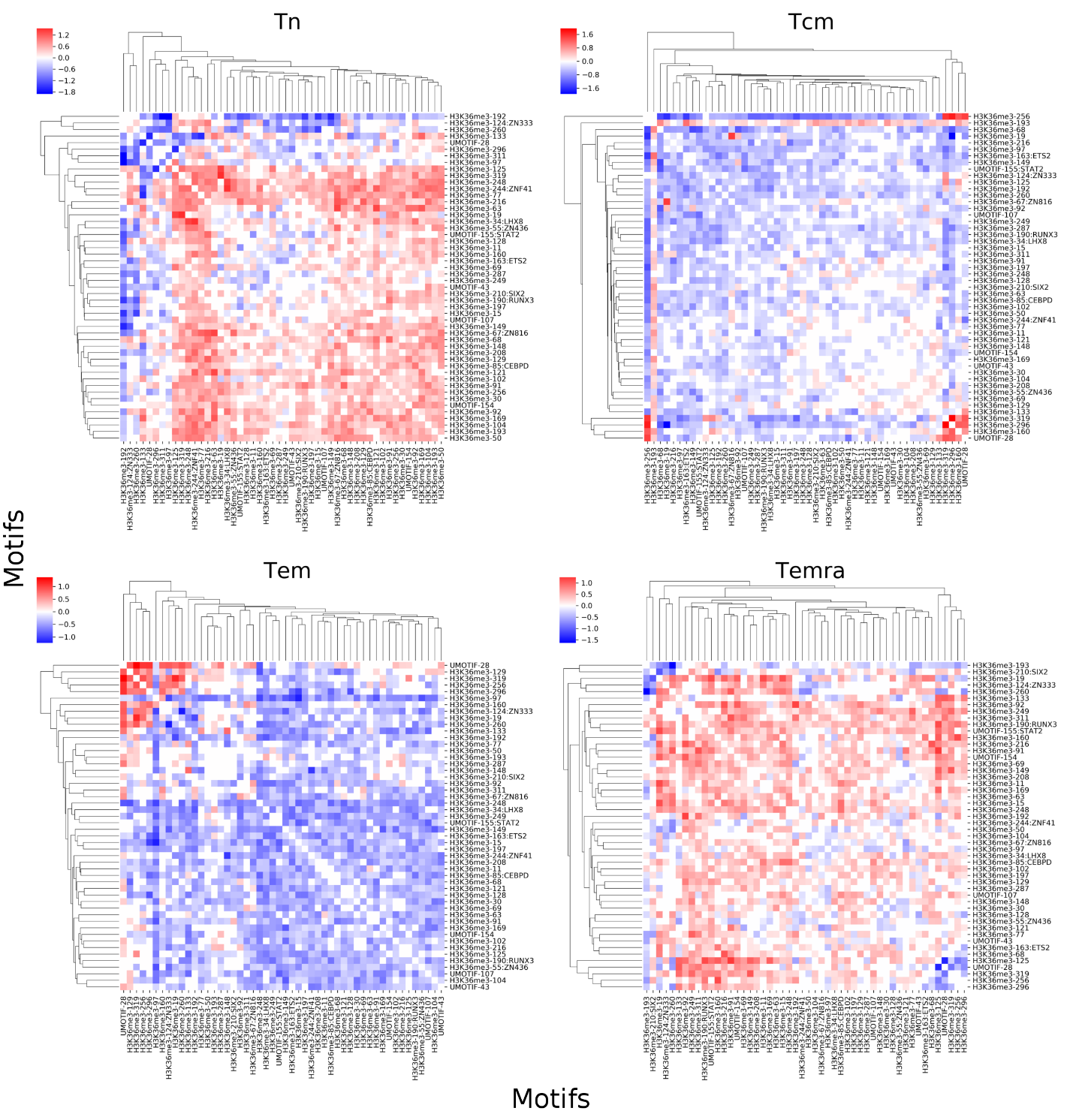


**Figure S13. Interactions between each pair of top 50 learned motifs on the prediction of the four cell types by the H3K36me3 model.** The heatmaps show the values of interaction coefficient γ between the top 50 learned motifs on predicting the indicated cell types in the H3K36me3 model. The scale bar shows range of interaction coefficient γ. A negative value indicates a negative interaction while a positive value indicates a positive interaction between the pair of motifs.
